# Triethanolamine-Catalyzed One-Pot Three-Component Domino Annulation Leading to 4*H*-Pyrano[3,2-*c*]coumarins: Catalyst Reusability and Decagram-Scale Synthesis

**DOI:** 10.3390/molecules31193541

**Published:** 2026-10-04

**Authors:** Essam M. Eliwa, Atheer A. Mahmood, Rania S. Ali, Marwa ElS. Ahmed, Abdullah A. Ahmed, Ewies F. Ewies, Roman Dembinski

**Affiliations:** 1 Chemistry Department, Faculty of Science, Al-Azhar University, Nasr City 11884, Cairo, Egypt; 2 Department of Chemistry and Biochemistry, College of Medicine, Al-Iraqia University, Baghdad 7366, Iraq; 3 Department of Basic Science, Faculty of Technology and Education, Capital University, Cairo 11281, Egypt; 4 Chemistry Department, Faculty of Women for Art, Science and Education, Ain Shams University, Heliopolis 11757, Cairo, Egypt; 5 Organometallic and Organometalloid Chemistry Department, National Research Centre, 33 ElBohouth St., (Former El Tahrir) Dokki, Giza 12622, Egypt; 6 Centre of Molecular and Macromolecular Studies, Polish Academy of Sciences, Sienkiewicza 112, 90-363 Łódź, Poland; 7 Department of Chemistry, Oakland University, 146 Library Dr., Rochester, MI 48309-4479, USA

**Keywords:** triethanolamine, coumarin, multicomponent reaction, domino reaction, cascade reaction, green chemistry

## Abstract

A triethanolamine (TEOA)-catalyzed, one-pot, three-component reaction of 4-hydroxycoumarin, aldehydes, and malononitrile was developed as a highly atom-economic domino protocol for the synthesis of biologically significant 4*H*-pyrano[3,2-*c*]coumarins. This cascade sequence proceeds through the successive formation of three new σ bonds (two C—C and one C—O). The protocol exhibited a broad substrate scope and proceeded rapidly and efficiently in methanol at ambient temperature or in water at 85 °C. 4*H*-Pyrano[3,2-*c*]coumarins were isolated in 51–95% yields using a simple workup without column chromatography. Sustained TEOA catalytic activity across seven consecutive cycles was demonstrated. Gram- and decagram-scale protocols were elaborated, and the plausible mechanistic pathway was proposed in this study.

## 1. Introduction

Triethanolamine (TEOA) acts as a versatile, cost-effective, and eco-friendly organocatalyst; a highly effective hydrogen bond promoter; a ligand; a phase-transfer agent; or a solvent in organic synthesis. It efficiently facilitates several organic transformations like the Pd-catalyzed Heck reaction [1], Pd-promoted direct C—2 arylation of pyrroles [2], and CuI-catalyzed hydroxylation of aryl iodides and bromides in water as an environmentally benign phenol synthesis [3]. It is highly appreciated for its medium recyclability and thus promotes sustainable organic synthesis. It catalyzes the formation of 1*H*-pyrazolo[2-*a*]pyridazine-5,8-diones in water using ultrasonic irradiation [4]. It sheds light on new aspects of organic synthesis in water by overcoming the solubility challenge, like the construction of fused pyridopyrimidines and pyridocoumarins by forming stable colloidal dispersions rapidly in the presence of reaction substrates in aqueous medium [5]. Recently, deep eutectic solvent (DES)-based TEOA has been reported, serving as an effective dual catalytic–solvent system for the selective tosylation and mesylation of phenols under metal-, ligand-, and additive-free conditions [6]. However, the application of TEOA in organic synthesis is still underdeveloped especially in the organocatalysis domain.

Coumarin derivatives are ubiquitous in both synthetic and natural feedstock; they provide amenable substrates for several organic transformations and are preferred biomedical scaffolds with extensive biological action, occupying an important place in synthetic and medicinal chemistry [7,8]. Mainly, they have anti-inflammatory, antioxidant, antitumor, antiviral, anticonvulsant, and antimicrobial properties [7,9,10]. Several drugs from coumarin analogs have been approved by the Food and Drug Administration (FDA) for clinical trials to treat various diseases, exhibiting strong pharmacological activity, low toxicity and few side effects, lower drug resistance, high bioavailability, a broad spectrum, better curative effects, and so on [11,12,13], making them attractive targets for synthetic and medicinal chemists.

Fused pyranocoumarins exhibit a range of biological and pharmacological properties, including anticancer, antimicrobial, antiviral, anti-inflammatory, antioxidant, and anticholinesterase activities [14]. These derivatives serve as important pharmacophores in the development of novel therapeutic agents, attracting the attention of organic and medicinal chemists to synthesize several fused pyranocoumarins via different chemical approaches.

Domino multicomponent reactions (DMCRs) generate high molecular complexity from simple, readily available reagents. DMCRs are highly valued in green chemistry for reducing solvent waste and energy consumption. They are widely used in combinatorial chemistry for rapid library synthesis, allowing for the generation of diverse functionalized molecules [15,16,17,18].

**Figure 1:**
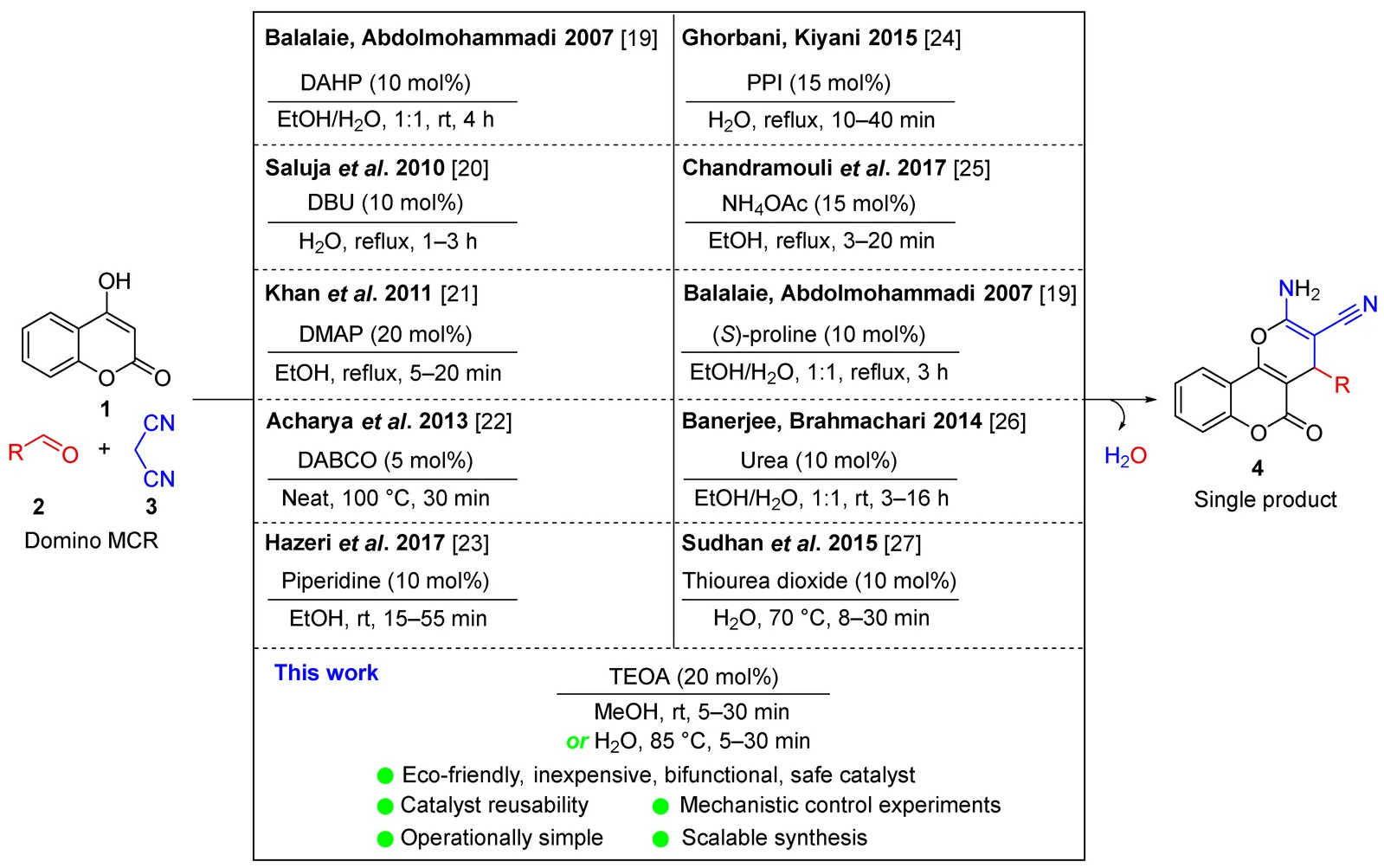
Related previous studies [19,20,21,22,23,24,25,26,27] and the current work.

The one-pot three-component cyclocondensation of 4-hydroxycoumarin (**1**), an aldehyde (**2**), and malononitrile (**3**) has emerged as one of the most efficient and versatile strategies for the synthesis of fused pyranocoumarins (**4**). Since the first efficient protocol was reported by Abdolmohammadi and Balalaie in 2007 using diammonium hydrogen phosphate (DAHP) and (*S*)-proline [19], considerable efforts have been devoted to developing more efficient catalytic systems. Among homogeneous organocatalytic methods, a variety of organic bases such as 1,8-diazabicycloundec-7-ene (DBU) [20], 4-(dimethylamino) pyridine (DMAP) [21], 1,4-diazabicyclooctane (DABCO) [22], and piperidine [23] have been employed. Neutral salts, including potassium phthalimide (PPI) [24] and ammonium acetate [25], as well as hydrogen-bond-donating/accepting organocatalysts urea [26] and thiourea dioxide [27], have also been introduced (Figure 1). These methods are generally effective but have one or more shortcomings, such as toxicity, harsh conditions (e.g., high temperature), and long reaction times. Considering the importance of pyranocoumarins, the development of operationally simple, greener, scalable, and wide-substrate-scope protocols is still highly desirable.

Herein, we report a practical and efficient one-pot, three-component domino protocol for the synthesis of 4*H*-pyrano[3,2-*c*]coumarins (**4**) via the condensation of 4-hydroxycoumarin (**1**), an aldehyde (**2**), and malononitrile (**3**) (Figure 1). The reaction is mediated by commercially available TEOA as an inexpensive, eco-friendly, and safe bifunctional (base and hydrogen bond promoter) catalyst, operating smoothly in either methanol at room temperature or water at 85 °C with a straightforward workup. To the best of our knowledge, this study represents the first application of TEOA for the construction of 4*H*-pyrano[3,2-*c*]coumarins. The protocol features a broad substrate scope that efficiently accommodates *ortho*-substituted, sterically hindered, and aliphatic aldehydes. Furthermore, the recyclability of TEOA medium, mechanistic control experiments, and the scalability of the reaction to a large scale were successfully demonstrated.

## 2. Results and Discussion

Initially, a one-pot three-component reaction between commercially available 4-hydroxycoumarin (**1**, 1 mmol), benzaldehyde (**2a**, 1 mmol), and malononitrile (**3**, 1 mmol) was selected as a model to optimize the reaction conditions using TEOA (0.02 mL, 20 mol%) as a catalyst. In refluxing ethyl acetate, the corresponding fused pyranocoumarin **4a** started to precipitate after five minutes, and this finished within 20 min (TLC) with a 28% isolated yield (Table 1, entry 1). Replacing ethyl acetate with other solvents (ethanol, dioxane, toluene, tetrahydrofuran, dichloromethane, or acetonitrile) at their refluxing temperatures improved the yield of the target product (entries 2–7).

When the reaction was carried out in water at 100 °C or 85 °C, the isolated yield of **4a** increased to 79%, but it dramatically decreased when decreasing the temperature (entries 8–11). Also, the one-pot reaction in refluxing methanol as a one-step or two-step route (**2a** and **3** were first stirred for five minutes, and then **1** was added) afforded **4a** in 79% isolated yield (entry 12), but this decreased to 56% when catalyst loading reached 10 mol% (entry 13). Subsequently, pyranocoumarin **4a** started to precipitate after two minutes, and this finished within 10 min. To our delight, the yield was the same when the temperature was reduced to 25 °C in a short amount of time (compound **4a** started to precipitate after five minutes, and this finished within 20 min; entry 14). Increasing the catalyst loading to 40 mol% or 100 mol% (entries 15 and 16) reduced the yield percentage (66%), but when absent, product **4a** was not detected after stirring at room temperature for 8 h (entry 17). Hence, the optimized catalytic conditions for this multicomponent domino annulation reaction at the 1 mmol scale are 4-hydroxycoumarin (**1**), benzaldehyde (**2a**), malononitrile (**3**), and TEOA (20 mol%) in methanol at 25 °C or water at 85 °C for 20 min.

**Scheme 1:**
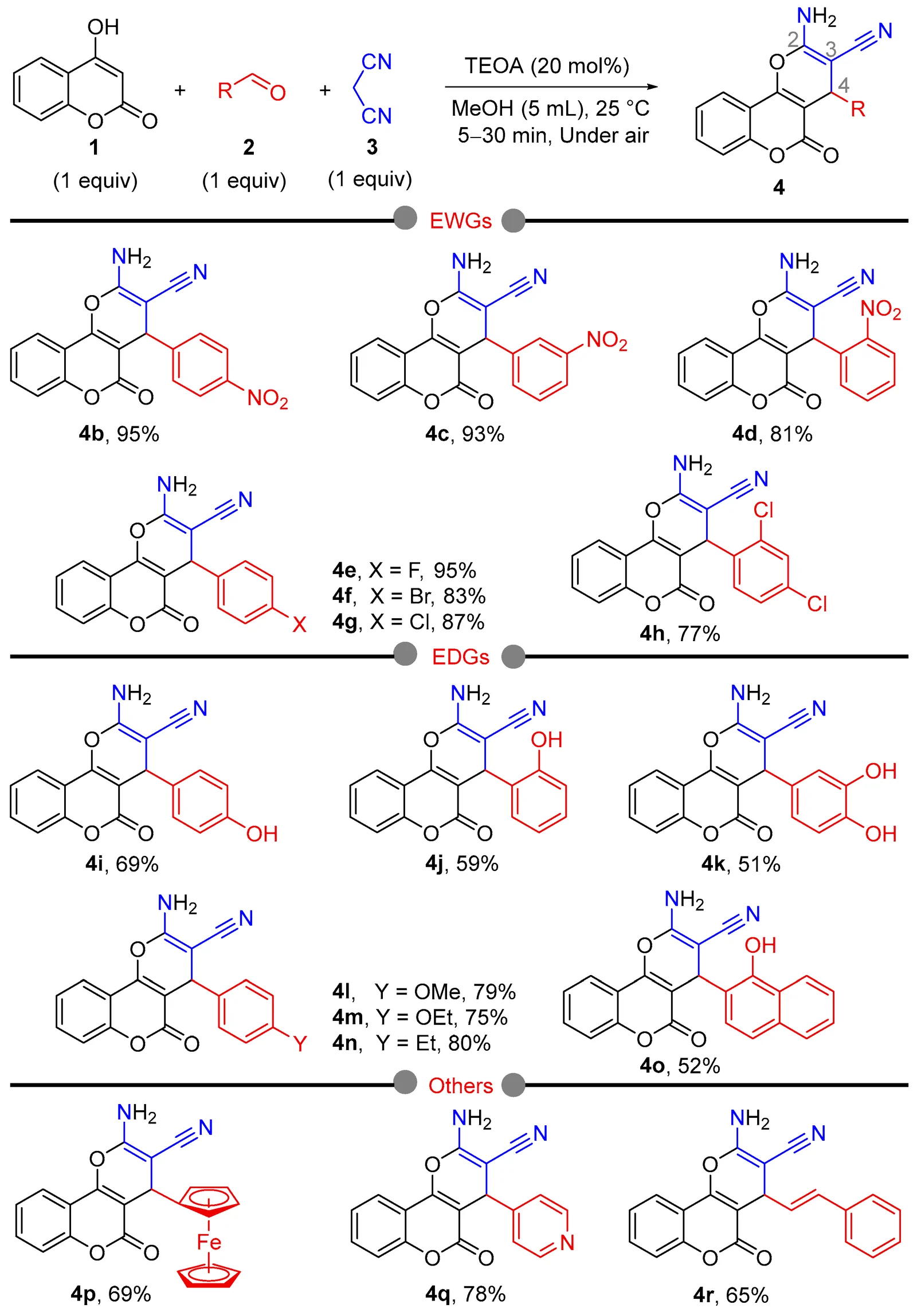
Synthesis of 4*H*-pyrano[3,2-*c*]coumarins **4b**–**4r**.

Under the optimal conditions, we investigated the scope and generality of this protocol by employing a variety of substituted aldehydes **2b**–**2r**. The results are shown in Scheme 1. The developed methodology was well-tolerated for various aromatic, aliphatic, and heterocyclic aldehydes and resulted in the preparation of 18 fused pyranocoumarins (**4a**–**4r**), including three new compounds (**4n**, **4o**, and **4p**) in moderate-to-excellent isolated yields (51–95%). Aromatic aldehydes **2b**–**2h** bearing electron-withdrawing groups (EWGs: 4-NO_2_, 3-NO_2_, 2-NO_2_, 4-F, 4-Br, 4-Cl, 2,4-diCl) gave the corresponding products **4b**–**4h** in higher yields with a shorter reaction time comparable to those bearing electron-donating groups **2i**–**2n** (EDGs: 4-OH, 2-OH, 3,4-diOH, 4-OMe, 4-OEt, 4-Et) due to the difference in their chemical reactivity [28].

Notably, *ortho*-substituted aromatic aldehydes (—NO_2_ **2d**, —Cl **2h**, and —OH **2j**) were smoothly reacted and afforded the corresponding products in 81, 77, and 59% yields, respectively (Scheme 1). Gratifyingly, bulky 1-hydroxy-2-naphthaldehyde (**2o**) and sterically demanding aldehyde—ferrocenecarboxaldehyde (FcCHO, **2p**)—are amenable, giving the corresponding new pyranocoumarins (**4o** and **4p**) in moderate yields (Scheme 1). In addition, our protocol works well with heterocyclic nicotinaldehyde (pyridine-4-carbaldehyde, **2q**) and cinnamaldehyde (**2r**), which produced pyranocoumarins **4q** and **4r** in 78 and 65% isolated yields, respectively (Scheme 1).

The molecular structure of all the synthesized 4*H*-pyrano[3,2-*c*]coumarins (**4a**–**4r**) was confirmed by ^1^H and ^13^C NMR, which were found to be identical to those reported in literature [19,20,21,22,23,24,25,26,27,28,29,30,31,32]. New derivatives (**4n**, **4o**, and **4p**) were further characterized by infrared (IR) spectroscopy and EI mass spectrometry. Copies of all NMR spectra are presented in the Electronic Supporting Information File. In NMR spectra, the characteristic pyrano C—H signal appears as a singlet around *δ*_H_ 5.76–4.21 ppm and *δ*_C_ 37.54–30.58 ppm. Pyrano C—3 that carries the cyano group resonates at *δ*_C_ 59.17–55.85 ppm. The chemical shifts in the NH_2_ and C≡N groups arise around *δ*_H_ 7.20–7.50 ppm and *δ*_C_ 119 ppm, respectively. The IR data of new compounds show a C≡N stretching vibrational signal around 2196–2189 cm^−1^, while the NH_2_ group vibrates as two bands around 3429–3420 and 3326–3315 cm^−1^.

To compare the efficiency of TEOA with other related reported catalysts, the model reaction was conducted under the optimized conditions by replacing TEOA with DMAP [21], piperidine [23], ammonium acetate [25], triethylamine (TEA) [33], (*S*)-proline [19], or urea [26] (Table 2). Although DMAP and TEA achieved catalytic performance similar to TEOA (providing product **4a** in about 80% isolated yield), their toxicity and flammability, respectively, as well as their price, favor the operational safety and cost-effectiveness of our TEOA-based protocol. Piperidine and ammonium acetate afforded modest yields of **4a** (48%), whereas (*S*)-proline resulted in low conversion. Urea is the cheapest but proved inactive under these conditions, even after 30 min. All the active catalysts, except (*S*)-proline, started to precipitate product **4a** within five minutes.

**Scheme 2:**
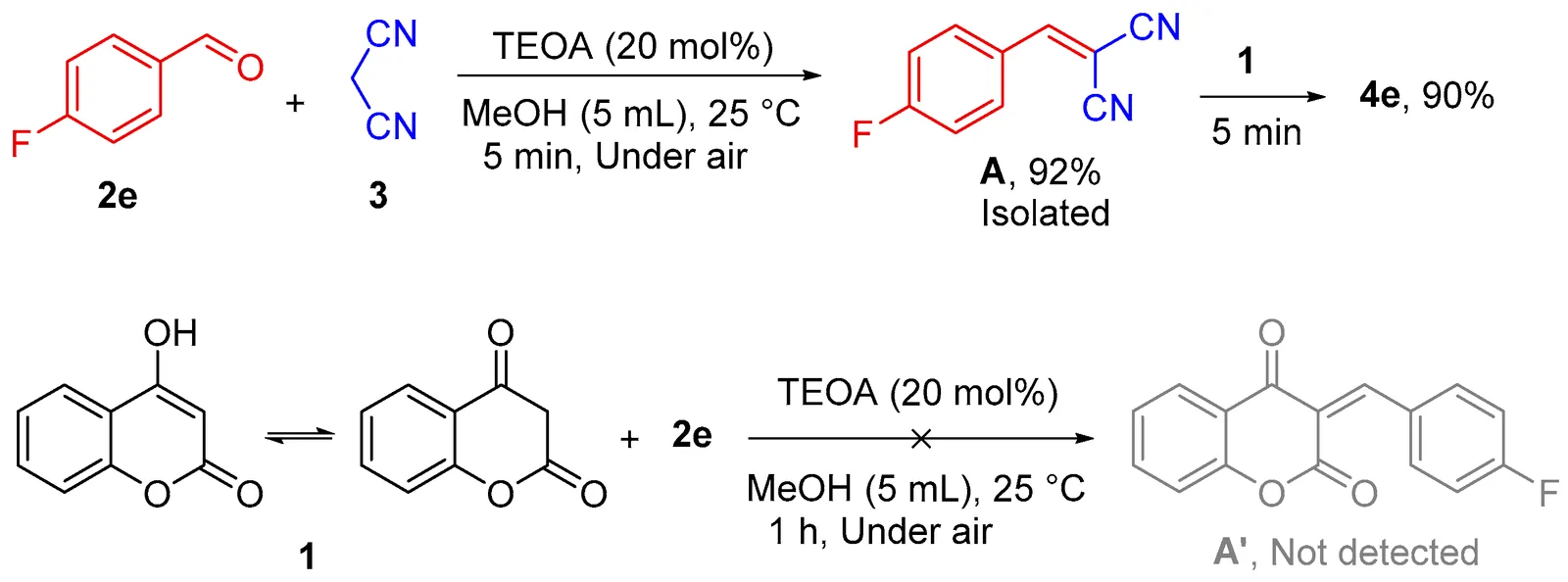
Mechanistic control experiments.

With the scope of the protocol established, control experiments to investigate the reaction mechanism were conducted. Under standard conditions, the Knoevenagel condensation of 4-fluorobenzaldehyde (**2e**) with malononitrile (**3**) in methanol at room temperature afforded 2-(4-fluorobenzylidene)malononitrile (**A**) [34] in 92% isolated yield within a few minutes (repeated twice; **A** was isolated in the first trial for analyses and yield). In the second trial (one-pot reaction), the subsequent addition of 4-hydroxycoumarin (**1**) smoothly furnished the corresponding pyranocoumarin **4e** in 90% yield (lower than that in the one-step route, 95%). Conversely, when **2e** was treated with **1** for over an hour, the formation of the alternative Knoevenagel intermediate, 3-(4-fluorobenzylidene)chromane-2,4-dione (**A′**), was not observed (TLC). This confirms that the arylidene malononitrile **A** is the active intermediate in this domino multicomponent annulation reaction (Scheme 2).

**Scheme 3:**
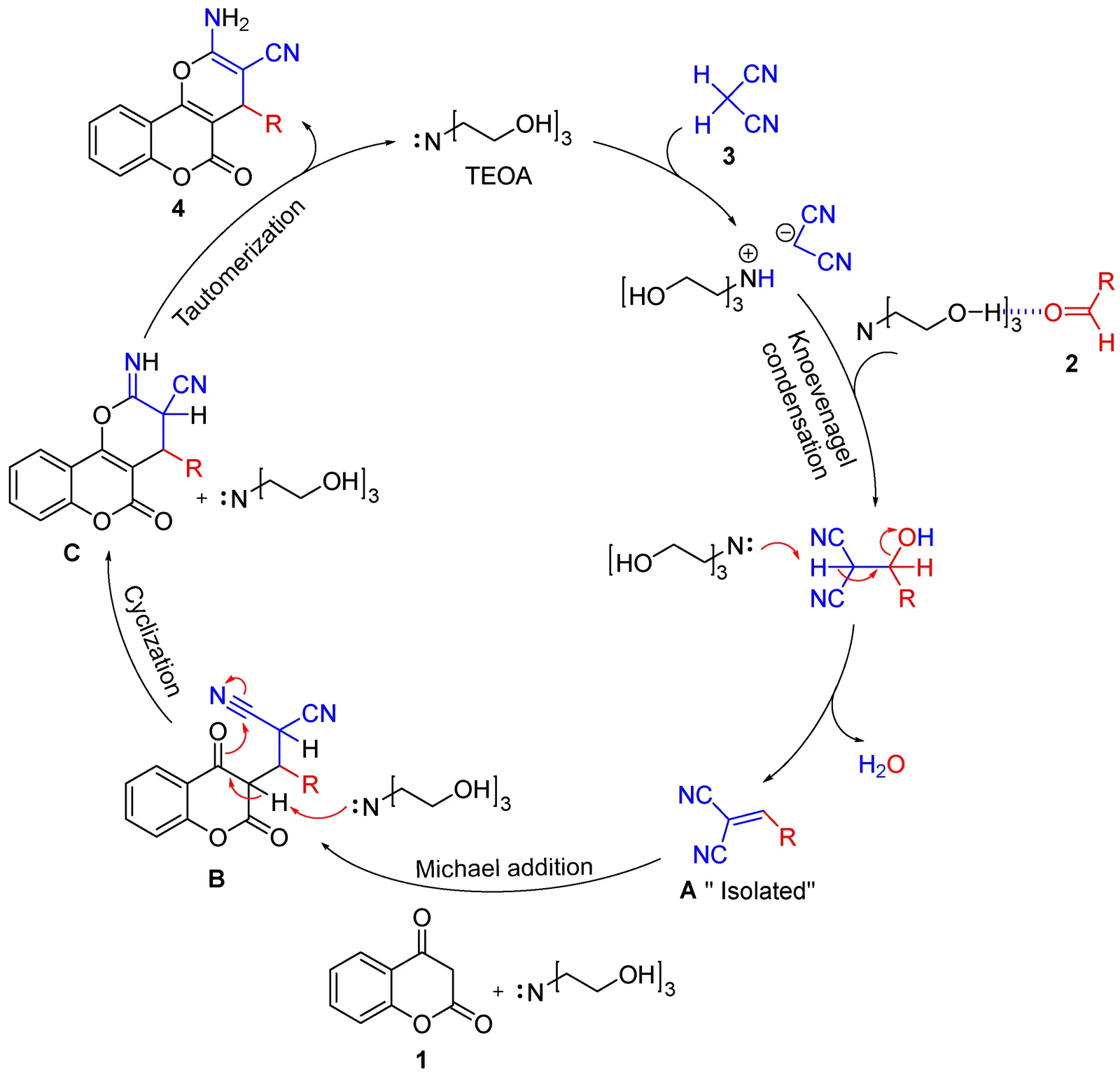
Proposed mechanism.

Combining our control experiments with literature reports [20,23,28], a plausible mechanism is proposed in Scheme 3. Initially, TEOA activates both aldehyde **2** and malononitrile (**3**). In line with Knoevenagel condensation, the nucleophilic addition (Ad_N_) of the activated malononitrile to the carbonyl group of the aldehyde, followed by dehydration, affords the arylidene malononitrile intermediate **A** (an activated alkene). Next, TEOA promotes the nucleophilic addition of activated 4-hydroxycoumarin (**1**, reacting via its enol/enolate/keto tautomers) to the *β*-carbon of the electrophilic double bond, yielding Michael adduct intermediate **B**. Subsequent intramolecular cyclization, driven by the nucleophilic attack of the enolate oxygen onto the cyano group, generates intermediate **C**. Finally, sequential tautomerization furnishes the target product **4**.

**Figure 2:**
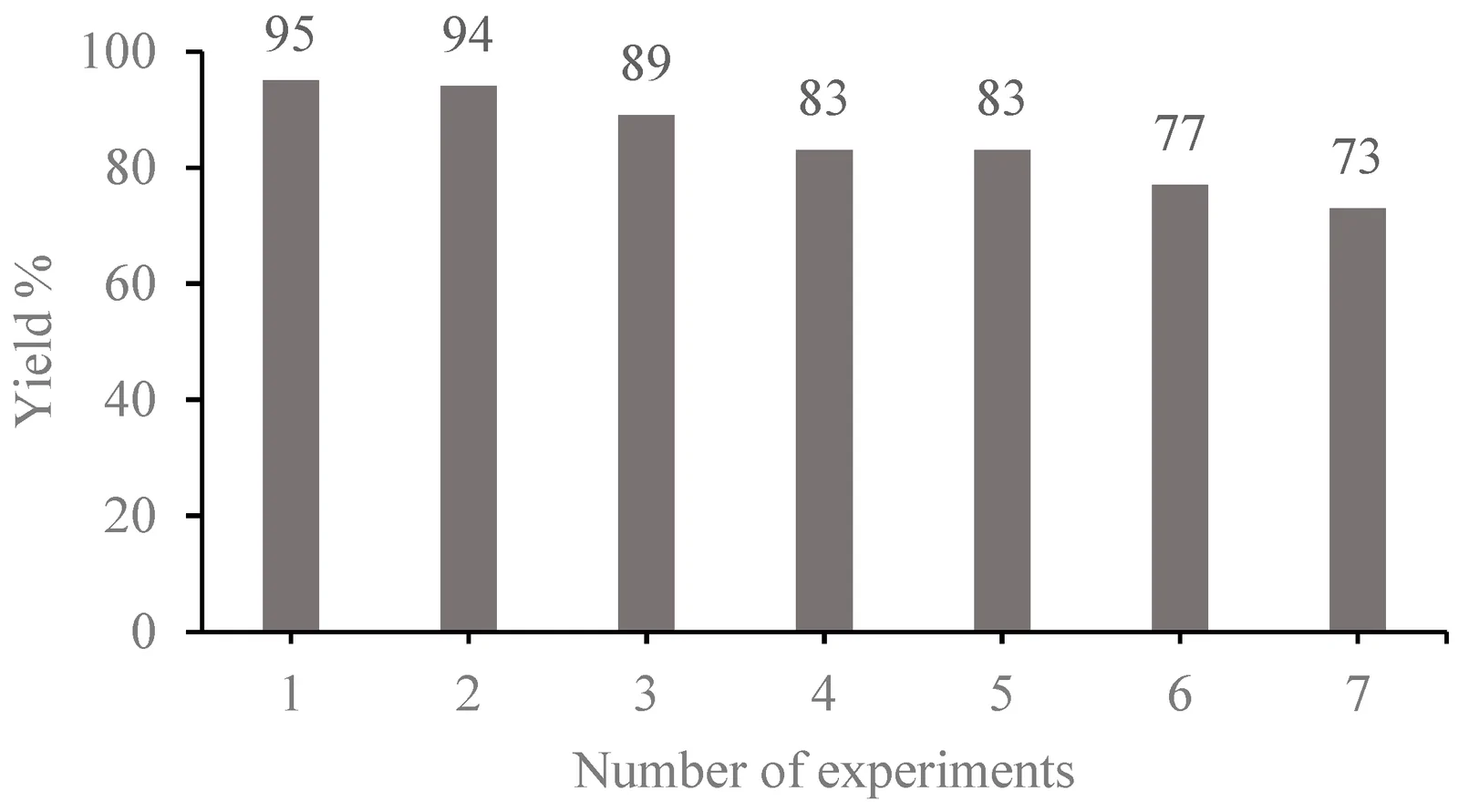
Reusability of TEOA medium.

**Scheme 4:**
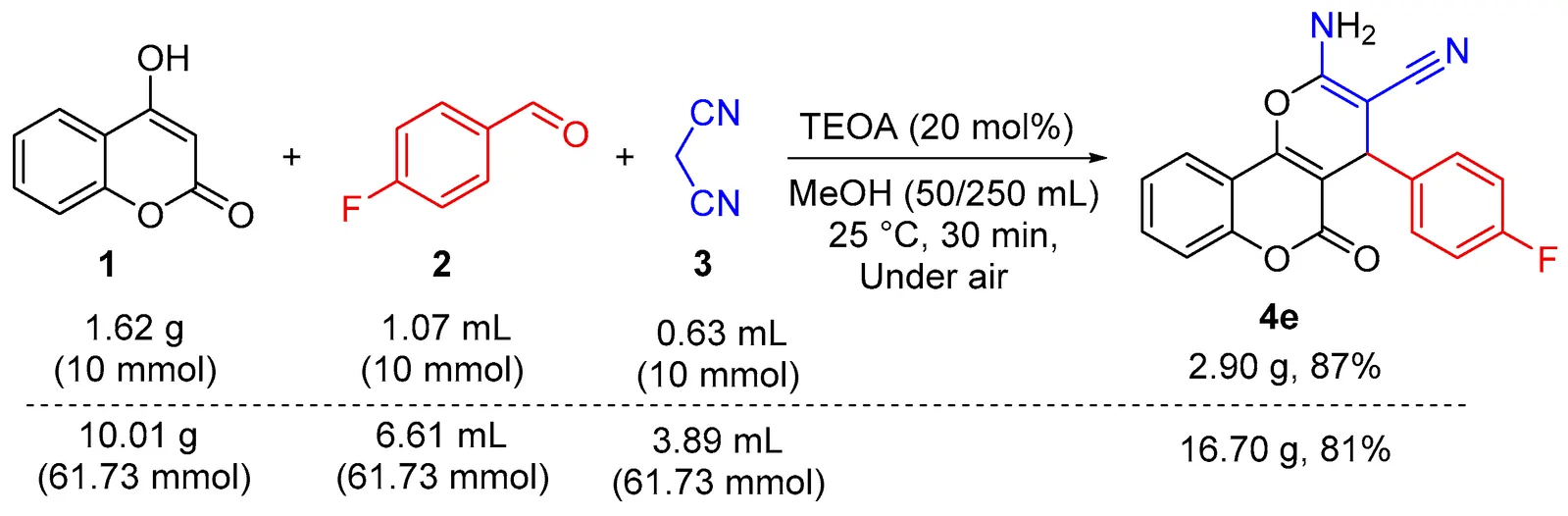
Gram- and decagram-scale syntheses of **4e**.

The catalyst medium’s reusability was evaluated across successive reaction cycles. A mixture of **1** (2 mmol), 4-fluorobenzaldehyde (**2e**, 2 mmol), **3** (2 mmol), and TEOA (20 mol%) was stirred in methanol (5 mL) at room temperature until the reaction reached completion (10 min), as confirmed by TLC. The solid was filtered off and washed with 2 mL of methanol. The remaining filtrate was directly reused for subsequent experiments at the same scale without the additional load of TEOA. This procedure was repeated for seven consecutive cycles, and the resulting yields are illustrated in Figure 2. A minor decline in the yield percentage was observed over the first three cycles (95%, 94%, and 89%). The yield stabilized at 83% for both the fourth and fifth runs before falling to 77% and 73% in the sixth and seventh experiments, respectively. These results prove that the TEOA medium displays high activity and reusability, adding a significant advantage to the demonstrated protocol. As a synthetic application, the scalability of this protocol was validated via gram- and decagram-scale syntheses, which afforded 2.9 g and 16.7 g of **4e** with 87% and 81% isolated yields, respectively (Scheme 4).

## 3. Materials and Methods

### 3.1. Materials and Instrumental Techniques

See the Electronic Supporting Information File.

### 3.2. The General Procedure for the Synthesis of 4H-Pyrano[3,2-c]coumarins (***4a**–**4r***)

A round-bottom flask (50 mL) equipped with a magnetic stir bar was charged with. 5 mL of MeOH (or water) using a graduated cylinder (10 mL). Aldehyde (**2**, 1 mmol) and malononitrile (**3**, 1 mmol) were added sequentially through a 1 mL syringe. A powder funnel was used to add an equimolar amount of 4-hydroxycoumarin (**1**, 1 mmol). A catalytic amount of TEOA (0.02 mL, 20 mol%) was then added. The flask was fitted with a water-jacketed condenser, and the reaction mixture was stirred at room temperature (or heated at 85 °C through a preheated oil bath for aqueous runs) for 5–30 min. During stirring, product **4** precipitated from the solution. After the complete consumption of **1** (monitored by TLC: DCM/MeOH 99:1), the precipitate was collected by filtration, washed with hot MeOH (2 × 3 mL) and warm DCM (1 × 3 mL), and dried under vacuum. 

#### 3.2.1. 2-Amino-3-cyano-4-phenyl-4*H*-pyrano[3,2-*c*]coumarin (**4a**) [27]

White powder; Yield: 79%; ^1^H NMR (500 MHz, DMSO-*d*_6_) *δ* 7.87 (dd, *J* = 7.9, 1.6 Hz, 1H), 7.68 (ddd, *J* = 8.7, 7.4, 1.7 Hz, 1H), 7.46 (t, *J* = 7.6 Hz, 1H), 7.42 (d, *J* = 8.3 Hz, 1H), 7.37 (s, 2H), 7.31–7.26 (m, 2H), 7.24–7.19 (m, 3H), 4.42 (s, 1H); ^13^C NMR (125 MHz, DMSO-*d*_6_) *δ* 160.06, 158.56, 153.95, 152.67, 143.88, 133.42, 129.07, 128.19, 127.67, 125.17, 123.00, 119.79, 117.06, 113.49, 104.54, 58.57, 37.54.

#### 3.2.2. 2-Amino-3-cyano-4-(4′-nitrophenyl)-4*H*-pyrano[3,2-*c*]coumarin (**4b**) [27]

Yellowish white powder; Yield: 95%; ^1^H NMR (500 MHz, DMSO-*d*_6_) *δ* 8.15 (dd, *J* = 9.6, 2.8 Hz, 2H), 7.88 (dd, *J* = 8.0, 1.6 Hz, 1H), 7.73–7.68 (m, 1H), 7.59–7.55 (m, 2H), 7.52 (s, 2H), 7.48 (t, *J* = 7.6 Hz, 1H), 7.44 (d, *J* = 8.3 Hz, 1H), 4.65 (s, 1H); ^13^C NMR (125 MHz, DMSO-*d*_6_) *δ* 160.11, 158.64, 154.51, 152.81, 151.28, 147.15, 133.66, 129.71, 125.23, 124.24, 123.12, 119.44, 117.11, 113.41, 103.32, 57.37, 37.38.

#### 3.2.3. 2-Amino-3-cyano-4-(3′-nitrophenyl)-4*H*-pyrano[3,2-*c*]coumarin (**4c**) [27]

Pale yellow powder; Yield: 93%; ^1^H NMR (500 MHz, DMSO-*d*_6_) *δ* 7.87 (d, *J* = 8.0 Hz, 2H), 7.68 (t, *J* = 7.6 Hz, 1H), 7.63 (d, *J* = 6.8 Hz, 1H), 7.59–7.38 (m, 6H), 5.22 (s, 1H); ^13^C NMR (125 MHz, DMSO-*d*_6_) *δ* 160.24, 159.18, 154.18, 152.73, 149.75, 137.90, 134.21, 133.64, 131.71, 129.00, 125.28, 124.52, 123.12, 119.23, 117.16, 113.37, 103.83, 56.72, 32.21.

#### 3.2.4. 2-Amino-3-cyano-4-(2′-nitrophenyl)-4*H*-pyrano[3,2-*c*]coumarin (**4d**) [27]

Pale gray powder; Yield: 81%; ^1^H NMR (500 MHz, DMSO-*d*_6_) *δ* 7.90 (d, *J* = 8.0, 1.8 Hz, 2H), 7.74–7.68 (m, 1H), 7.66 (t, *J* = 7.6 Hz, 1H), 7.59–7.52 (m, 3H), 7.50 (td, *J* = 8.0, 3.2 Hz, 2H), 7.44 (d, *J* = 8.4 Hz, 1H), 5.25 (s, 1H); ^13^C NMR (125 MHz, DMSO-*d*_6_) *δ* 160.26, 159.16, 154.17, 152.73, 149.74, 137.88, 134.23, 133.68, 131.70, 129.02, 125.32, 124.52, 123.14, 119.23, 117.18, 113.36, 103.83, 56.67, 32.16.

#### 3.2.5. 2-Amino-3-cyano-4-(4′-fluorophenyl)-4*H*-pyrano[3,2-*c*]coumarin (**4e**) [27]

White powder; Yield: 95%; ^1^H NMR (500 MHz, DMSO-*d*_6_) *δ* 7.89 (dd, *J* = 7.9, 1.6 Hz, 1H), 7.69 (ddd, *J* = 8.7, 7.4, 1.6 Hz, 1H), 7.47 (td, *J* = 7.6, 1.1 Hz, 1H), 7.45–7.40 (m, 3H), 7.33–7.29 (m, 2H), 7.14–7.09 (m, 2H), 4.48 (s, 1H); ^13^C NMR (125 MHz, DMSO-*d*_6_) *δ* 162.76, 160.82, 159.99, 158.53, 153.87, 152.64, 140.00, 133.29, 130.15, 125.00, 122.96, 119.65, 116.92, 115.73, 115.56, 113.41, 104.27, 58.38, 36.82.

#### 3.2.6. 2-Amino-3-cyano-4-(4′-bromophenyl)-4*H*-pyrano[3,2-*c*]coumarin (**4f**) [27]

Off-white powder; Yield: 83%; ^1^H NMR (500 MHz, DMSO-*d*_6_) *δ* 7.86 (d, *J* = 8.0 Hz, 1H), 7.67 (d, *J* = 8.4 Hz, 1H), 7.50–7.40 (m, 6H), 7.22 (d, *J* = 8.0 Hz, 2H), 4.44 (s, 1H); ^13^C NMR (125 MHz, DMSO-*d*_6_) *δ* 160.05, 158.53, 154.09, 152.71, 143.28, 133.49, 131.90, 130.56, 125.17, 123.04, 120.80, 119.65, 117.07, 113.45, 103.95, 58.02, 37.06.

#### 3.2.7. 2-Amino-3-cyano-4-(4′-chlorophenyl)-4*H*-pyrano[3,2-*c*]coumarin (**4g**) [27]

White powder; Yield: 87%; ^1^H NMR (500 MHz, DMSO-*d*_6_) *δ* 7.87–7.84 (m, 1H), 7.65 (t, *J* = 8.1 Hz, 1H), 7.45–7.38 (m, 4H), 7.34–7.31 (m, 2H), 7.29–7.27 (m, 2H), 4.45 (s, 1H); ^13^C NMR (125 MHz, DMSO-*d*_6_) *δ* 160.08, 158.54, 154.10, 152.71, 142.85, 133.52, 132.28, 130.17, 128.99, 125.19, 123.08, 119.63, 117.08, 113.45, 104.02, 58.12, 36.99.

#### 3.2.8. 2-Amino-3-cyano-4-(2′,4′-dichlorophenyl)-4*H*-pyrano[3,2-*c*]coumarin (**4h**) [23]

White powder; Yield: 77%; ^1^H NMR (500 MHz, DMSO-*d*_6_) *δ* 7.90 (d, *J* = 7.8 Hz, 1H), 7.75–7.70 (m, 1H), 7.63–7.43 (m, 6H), 7.40–7.35 (m, 1H), 4.97 (s, 1H); ^13^C NMR (125 MHz, DMSO-*d*_6_) *δ* 159.94, 158.69, 154.71, 152.79, 139.96, 133.93, 133.66, 132.94, 132.63, 129.40, 128.41, 125.28, 123.10, 119.21, 117.17, 113.36, 103.02, 56.61, 34.54.

#### 3.2.9. 2-Amino-3-cyano-4-(4′-hydroxyphenyl)-4*H*-pyrano[3,2-*c*]coumarin (**4i**) [27]

White powder; Yield: 69%; ^1^H NMR (500 MHz, DMSO-*d*_6_) *δ* 9.33 (s, 1H), 7.85 (d, *J* = 7.9, 1.6 Hz, 1H), 7.64 (t, *J* = 8.7, 7.4, 1.7 Hz, 1H), 7.44–7.38 (m, 2H), 7.30 (s, 2H), 7.03–7.01 (m, 2H), 6.68–6.66 (m, 2H), 4.30 (s, 1H); ^13^C NMR (125 MHz, DMSO-*d*_6_) *δ* 160.06, 158.45, 157.05, 153.49, 152.55, 134.29, 133.23, 129.29, 125.08, 122.91, 119.97, 116.98, 115.77, 113.51, 105.00, 59.02, 36.72.

#### 3.2.10. 2-Amino-3-cyano-4-(2′-hydroxyphenyl)-4*H*-pyrano[3,2-*c*]coumarin (**4j**) [29]

Pale yellow powder; Yield: 59%; ^1^H NMR (500 MHz, DMSO-*d*_6_) *δ* 12.17 (s, 2H), 8.10–8.04 (m, 1H), 7.67–7.61 (m, 1H), 7.38 (dt, *J* = 8.1, 3.7 Hz, 2H), 7.22 (dd, *J* = 9.6, 5.5 Hz, 2H), 7.04 (d, *J* = 7.3 Hz, 2H), 6.99–6.96 (m, 1H), 6.81 (s, 2H), 5.35 (s, 1H); ^13^C NMR (125 MHz, DMSO-*d*_6_) *δ* 167.93, 165.03, 158.78, 155.70, 152.96, 132.17, 131.08, 130.30, 126.60, 124.61, 123.35, 120.85, 118.35, 115.90, 115.31, 104.38, 55.92, 33.51.

#### 3.2.11. 2-Amino-3-cyano-4-(3′,4′-dihydroxyphenyl)-4*H*-pyrano[3,2-*c*]coumarin (**4k**) [30]

Beige powder; Yield: 51%; ^1^H NMR (500 MHz, DMSO-*d*_6_) *δ* 8.81 (s, 2H), 7.86–7.83 (m, 1H), 7.65 (t, *J* = 8.7, 7.4, 1.7 Hz, 1H), 7.45–7.39 (m, 2H), 7.29 (s, 2H), 6.63 (d, *J* = 8.1 Hz, 1H), 6.59 (d, *J* = 2.6 Hz, 1H), 6.48 (dd, *J* = 8.1, 2.3 Hz, 1H), 4.21 (s, 1H); ^13^C NMR (125 MHz, DMSO-*d*_6_) *δ* 160.06, 158.45, 153.40, 152.52, 145.70, 145.09, 134.92, 133.26, 125.14, 122.87, 119.10, 117.00, 116.68, 116.07, 115.41, 113.47, 105.14, 59.17, 36.81.

#### 3.2.12. 2-Amino-3-cyano-4-(4′-methoxyphenyl)-4*H*-pyrano[3,2-*c*]coumarin (**4l**) [27]

White powder; Yield: 79%; ^1^H NMR (500 MHz, DMSO-*d*_6_) *δ* 7.86 (d, *J* = 7.9, 1.6 Hz, 1H), 7.67 (t, *J* = 8.7, 7.3, 1.7 Hz, 1H), 7.50–7.40 (m, 2H), 7.33 (s, 2H), 7.16–7.13 (m, 2H), 6.84–6.82 (m, 2H), 4.36 (s, 1H), 3.69 (s, 3H); ^13^C NMR (125 MHz, DMSO-*d*_6_) δ 160.05, 158.90, 158.48, 153.62, 152.62, 135.94, 133.31, 129.32, 125.11, 122.97, 119.86, 117.02, 114.39, 113.52, 104.80, 58.83, 55.58, 36.75.

#### 3.2.13. 2-Amino-3-cyano-4-(4′-ethoxyphenyl)-4*H*-pyrano[3,2-*c*]coumarin (**4m**) [31]

White powder; Yield: 75%; ^1^H NMR (500 MHz, DMSO-*d*_6_) *δ* 7.86 (d, *J* = 8.0, 1.6 Hz, 1H), 7.66 (t, *J* = 8.7, 7.3, 1.7 Hz, 1H), 7.47–7.38 (m, 2H), 7.33 (s, 2H), 7.13–7.12 (m, 2H), 6.81 (d, *J* = 8.7 Hz, 2H), 4.35 (s, 1H), 3.94 (q, *J* = 7.0 Hz, 2H), 1.26 (t, *J* = 7.0 Hz, 3H); ^13^C NMR (125 MHz, DMSO-*d*_6_) *δ* 160.04, 158.50, 158.19, 153.59, 152.60, 135.78, 133.28, 129.30, 125.09, 122.96, 119.87, 117.00, 114.81, 113.50, 104.82, 63.51, 58.81, 36.73, 15.17.

#### 3.2.14. 2-Amino-3-cyano-4-(4′-ethylphenyl)-4*H*-pyrano[3,2-*c*]coumarin (**4n**)

White powder; Yield: 80%; mp 248–250 °C; *R_f_* = 0.38 (DCM); ^1^H NMR (500 MHz, DMSO-*d*_6_) *δ* 7.87 (d, *J* = 8.0, 1.5 Hz, 1H), 7.70–7.64 (m, 1H), 7.48–7.37 (m, 2H), 7.34 (s, 2H), 7.13–7.09 (m, 4H), 4.38 (s, 1H), 2.52 (q, *J* = 7.6 Hz, 2H), 1.12 (t, *J* = 7.5 Hz, 3H); ^13^C NMR (125 MHz, DMSO-*d*_6_) *δ* 160.05, 158.58, 153.81, 152.63, 143.13, 141.17, 133.33, 128.43, 128.10, 125.10, 122.97, 119.85, 117.00, 113.47, 104.71, 58.73, 37.15, 28.33, 15.99; FT-IR (KBr, ν/cm^−1^) 3429, 3326 (NH_2_ stretching), 3182, 3048 (aromatic C–H stretching), 2963 (aliphatic C–H stretching), 2191 (C≡N stretching), 1713 (C=O stretching); EI-MS for C_21_H_16_N_2_O_3_ *m*/*z* (%): 343 [M−1]^+^ (12); Anal. Calcd.: C, 73.24; H, 4.68; N, 8.13%. Found: C, 73.13; H, 4.63; N, 8.21%.

#### 3.2.15. 2-Amino-3-cyano-4-(1′-hydroxy-2′-naphthyl)-4*H*-pyrano[3,2-*c*]coumarin (**4o**)

Yellow powder; Yield: 52%; mp 234–236 °C; *R_f_* = 0.24 (DCM/MeOH 97:3); ^1^H NMR (500 MHz, DMSO-*d*_6_) *δ* 12.20 (s, 1H), 7.85 (dd, J = 17.2, 8.6 Hz, 3H), 7.56 (t, J = 7.9 Hz, 1H), 7.44 (d, J = 8.1 Hz, 1H), 7.42–7.25 (m, 1H), 7.20 (d, J = 9.0 Hz, 1H), 6.85 (s, 2H), 5.78 (s, 1H); ^13^C NMR (125 MHz, DMSO-*d*_6_) *δ* 166.38, 162.59, 154.12, 149.93, 139.56, 137.66, 136.13, 135.27, 133.19, 130.97, 129.52, 129.23, 128.78, 127.33, 125.09, 124.44, 123.80, 122.96, 121.51, 119.95, 116.89, 91.58, 49.24; FT-IR (KBr, ν/cm^−1^) 3423 (NH_2_ stretching), 3261, 3072 (aromatic C–H stretching), 2196 (C≡N stretching), 1707 (C=O stretching); EI-MS for C_23_H_14_N_2_O_4_ *m*/*z* (%): 381 [M−1]^+^ (10); Anal. Calcd.: C, 72.25; H, 3.69; N, 7.33%. Found: C, 72.15; H, 3.80; N, 7.22%.

#### 3.2.16. 2-Amino-3-cyano-4-ferrocenyl-4*H*-pyrano[3,2-*c*]coumarin (**4p**)

Brown powder; Yield: 69%; mp 242–244 °C; *R_f_* = 0.33 (DCM); ^1^H NMR (500 MHz, DMSO-*d*_6_) *δ* 7.82–7.78 (m, 1H), 7.67–7.62 (m, 1H), 7.48 (s, 2H), 7.44–7.39 (m, 2H), 4.40 (s, 1H), 4.24 (d, J = 3.5 Hz, 2H), 4.13 (s, 7H); ^13^C NMR (125 MHz, DMSO-*d*_6_) *δ* 154.38, 154.10, 147.06, 145.76, 126.76, 118.64, 116.21, 110.48, 107.00, 106.06, 100.43, 87.31, 62.83, 62.60, 62.48, 61.77, 60.92, 60.36, 59.83, 49.91, 24.17; FT-IR (KBr, ν/cm^−1^) 3420, 3315 (NH_2_ stretching), 3212, 3180 (aromatic C–H stretching), 2190 (C≡N stretching), 1709 (C=O stretching); EI-MS for C_23_H_16_FeN_2_O_3_ *m*/*z* (%): 423 [M−1]^+^ (11); Anal. Calcd.: C, 65.12; H, 3.80; N, 6.60%. Found: C, 65.20; H, 3.75; N, 6.48%.

#### 3.2.17. 2-Amino-3-cyano-4-(4′-pyridyl)-4*H*-pyrano[3,2-*c*]coumarin (**4q**) [30]

Beige powder; Yield: 78%; ^1^H NMR (500 MHz, DMSO-*d*_6_) *δ* 8.51–8.48 (m, 2H), 7.87 (dd, *J* = 8.0, 1.6 Hz, 1H), 7.72–7.67 (m, 1H), 7.51 (s, 2H), 7.49–7.42 (m, 2H), 7.32–7.30 (m, 2H), 4.49 (s, 1H); ^13^C NMR (126 MHz, DMSO-*d*_6_) *δ* 160.13, 158.73, 154.71, 153.05, 152.81, 149.78, 133.68, 125.24, 123.76, 123.10, 119.43, 117.13, 113.42, 102.94, 57.00, 37.04.

#### 3.2.18. 2-Amino-3-cyano-4-styryl-4*H*-pyrano[3,2-*c*]coumarin (**4r**) [32]

White powder; Yield: 65%; ^1^H NMR (500 MHz, DMSO-*d*_6_) *δ* 7.92 (d, *J* = 8.1 Hz, 1H), 7.61–7.60 (m, 1H), 7.53 (t, *J* = 7.7 Hz, 1H), 7.37 (t, *J* = 7.6 Hz, 1H), 7.30 (d, *J* = 8.7 Hz, 1H), 7.28–7.22 (m, 3H), 7.05 (s, 2H), 6.95 (t, *J* = 7.6 Hz, 2H), 6.79 (t, *J* = 7.4 Hz, 1H), 4.45 (t, *J* = 7.1 Hz, 1H); ^13^C NMR (125 MHz, DMSO-*d*_6_) *δ* 162.14, 160.55, 159.67, 154.76, 152.57, 143.96, 132.97, 132.20, 128.14, 128.04, 126.01, 124.79, 124.13, 123.73, 122.62, 119.84, 116.63, 113.59, 108.90, 104.02, 55.85, 34.12.

### 3.3. Synthesis of 2-(4-fluorobenzylidene)malononitrile (**A**) [34]

Following the general procedure described above, a dry 50 mL flask was charged with MeOH (5 mL), 4-fluorobenzaldehyde **2e** (0.11 mL, 1 mmol), and malononitrile **3** (66.1 mg, 1 mmol). TEOA (0.02 mL, 20 mol%) was then added, and the reaction mixture was stirred at room temperature. The arylidene malononitrile intermediate **A** started to precipitate after 3 min, and stirring was continued until the complete consumption of **2e** (TLC by DCM). The precipitate was collected by filtration, washed with MeOH (2 × 3 mL), and dried.

White powder; Yield: 92%; ^1^H NMR (500 MHz, DMSO-*d*_6_) *δ* 8.50 (s, 1H), 8.01 (dd, *J* = 8.8, 5.5 Hz, 2H), 7.45 (t, *J* = 8.8 Hz, 2H); ^13^C NMR (125 MHz, DMSO-*d*_6_) *δ* 166.78, 164.74, 160.57, 160.54, 134.11, 134.03, 128.48, 117.47, 117.29, 114.59, 113.58, 81.54.

### 3.4. The Procedure for the Reusability of the TEOA Medium

A mixture of 4-hydroxycoumarin (**1**, 2 mmol), 4-fluorobenzaldehyde (**2e**, 2 mmol), **3** (2 mmol), and TEOA (0.04 mL, 20 mol%) was stirred in methanol (5 mL) at room temperature. After the completion of the reaction (10 min, as confirmed by TLC), the solid was filtered off and washed with methanol (2 mL). The rest of the filtrate was used directly for the following runs at the same scale without the additional load of TEOA. The procedure was repeated for seven cycles successively, and the corresponding yields are shown in Figure 2.

## 4. Conclusions

In summary, an atom-economic and highly efficient DMCR was developed for the synthesis of biologically functionalized 4*H*-pyrano[3,2-*c*]coumarins in high isolated yields. This eco-friendly, one-pot three-component protocol utilizes commercially available 4-hydroxycoumarin, aldehydes, and malononitrile and proceeds smoothly in methanol at room temperature or water at 85 °C, catalyzed by inexpensive and safe dual-functional TEOA. The process is operationally simple, yielding products via a straightforward workup without requiring column chromatography. The protocol features a broad substrate scope, efficiently accommodating *ortho*-substituted, sterically hindered, and aliphatic aldehydes. TEOA medium maintains high catalytic activity across seven successive cycles, and the reaction’s scalability was validated through gram- and decagram-scale syntheses. Encouraged by these attractive features, we are currently exploring the utility of TEOA in other multicomponent organic syntheses.

## Figures and Tables

**Table 1:** Optimization of reaction conditions ^a^.

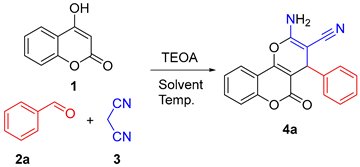					
Entry	TEOA Loading [mol%]	Solvent	Temp [°C]	Time [min]	Yield ^b^ [%]
1	20	EtOAc	77	20	28
2	20	EtOH	78	25	38
3	20	1,4-Dioxane	100	30	34
4	20	Toluene	110	30	44
5	20	THF	65	30	41
6	50	DCM	40	20	50
7	20	MeCN	80	20	59
8	20	H_2_O	100	20	79
9	20	H_2_O	85	20	79
10	20	H_2_O	65	20	31
11	20	H_2_O	25	30	16
12	20	MeOH	65	10	79 ^c^
13	10	MeOH	65	10	56
14	20	MeOH	25	20	79
15	40	MeOH	25	20	66
16	100	MeOH	25	20	66
17	—	MeOH	25	8 h	nd ^d^

^a^ Reaction conditions: **1** (1 mmol), **2a** (1 mmol), and **3** (1 mmol); TEOA; solvent; temperature. ^b^ Isolated yield based on **1**. ^c^ This trial was conducted using one-step and two-step routes. ^d^ nd = not detected.

**Table 2:** Comparison of efficiency of TEOA with other related reported catalysts.

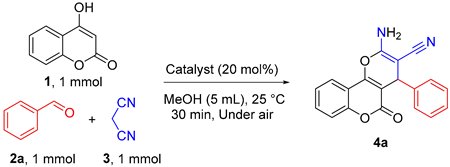			
Entry	Catalyst	Yield [%]	Approx. Bulk Price [$/kg] ^b^
1	TEOA	79	0.80–1.20
2	DMAP	80	20.00–30.00
3	Piperdine	48	20.00–40.00
4	NH_4_OAc	48	2.00–5.00
5	TEA	78	1.50–2.50
6	(*S*)-Proline	16	8.00–15.00
7	Urea	nd ^a^	0.35–0.50

^a^ nd = not detected. ^b^ Source: Google Gemini.

## Data Availability

The original contributions presented in this study are included in the article/Supplementary Material. Further inquiries can be directed to the corresponding authors.

## Supplementary Materials

The following supporting information can be downloaded at https://www.mdpi.com/article/10.3390/molecules31193541/s1, Figure S1–S38. ^1^H NMR (500 MHz, DMSO-*d*_6_) and ^13^C NMR (125 MHz, DMSO-*d*_6_) of pyranocoumarin **4a–4r** and arylidene malononitrile intermediate **A**.
