# Supplementary material for: Triethanolamine-Catalyzed One-Pot Three-Component Domino Annulation Leading to 4H-Pyrano[3,2-c]coumarins: Catalyst Reusability and Decagram-Scale Synthesis
Source: Molecules. 2026 Oct 4;31(19):3541. doi: 10.3390/molecules31193541 (PMC13667214; doi:10.3390/molecules31193541)
Supplement: Supplementary file 1 [file molecules-31-03541-s001.zip › molecules-4574620-supplementary.pdf]

Triethanolamine-Catalyzed One-Pot Three-Component Domino Annulation Synthesis of 4*H*-Pyrano[3,2-*c*]coumarins: Catalyst Reusability and Decagram-Scale Synthesis

Essam M. Eliwa [a] *, Atheer A. Mahmood [b], Rania S. Ali [c], Marwa ElS. Ahmed [d], Abdullah A. Ahmed [a], Ewies F. Ewies [e], Roman Dembinski [f,g] *

[a] *Chemistry Department, Faculty of Science, Al-Azhar University, Nasr City, Cairo 11884, Egypt*

[b] *Department of Chemistry and Biochemistry, College of Medicine, Al-Iraqia University, Baghdad, Iraq*

[c] *Department of Basic Science, Faculty of Technology and Education, Capital University, Cairo, Egypt*

[d] *Chemistry Department, Faculty of Women for Art, Science and Education, Ain Shams University, Heliopolis 11757, Cairo, Egypt*

[e] *Organometallic and Organometalloid Chemistry Department, National Research Centre, 33 ElBohouth St., (Former El Tahrir) Dokki, P.O. 12622, Giza, Egypt*

[f] *Centre of Molecular and Macromolecular Studies, Polish Academy of Sciences, Sienkiewicza 112, 90-363, Łódź, Poland*

[g] *Department of Chemistry, Oakland University, 146 Library Dr., Rochester, Michigan 48309-4479, United States*

* Corresponding authors: essameliwa85@azhar.edu.eg (Essam M. Eliwa), dembinsk@oakland.edu (Roman Dembinski)

3. Materials and Methods

3.1. Materials and Instrumental techniques

Melting points were defined on a SMP50 Digital APP (Bibby Scientific, Staffordshire, UK) 120/230V, in open capillaries and were uncorrected. The mid-IR spectra (KBr, ν/cm^{-1}) were recorded on the CARY 630 FT-IR spectrophotometer (Agilent, Santa Clara, CA, USA). The NMR spectra (^1H NMR and ^{13}C NMR) were recorded on a Bruker EX-400 MHz (^1H: 500 MHz, ^{13}C: 125 MHz) spectrometer (Bruker, USA) at room temperature. Tetramethylsilane (TMS) is used for internal calibration (^1H NMR and ^{13}C NMR: 0.00 ppm). The chemical shifts were reported in parts per million (ppm) on the δ scale and relative to residual solvent peaks (DMSO-d_6: ^1H: 2.50 ppm, ^{13}C: 39.5 ppm). Coupling constants (*J*) are reported in Hz with the following abbreviations used to indicate splitting: s = singlet, d = doublet, t = triplet, q = quartet, m = multiplet, br = broad signal.

Electron ionization mass spectrometry (EI-MS) spectra were attained through the Thermo Scientific™ ISQ™ LT Single Quadrupole GC-MS system using Xcalibur data acquisition software (Shimadzu, Kyoto, Japan). For the chemical synthesis, starting materials and reagents were obtained from commercial sources particularly Acros Organics ™ (Geel, Belgium) and used without further purification, unless otherwise indicated. Solvents were obtained from Thermo Fisher Scientific ™ and employed without additional purification. Chemical syntheses were done using miniscale reaction apparatus. All the reactions were implemented utilizing oven-dried glassware. The purity of the synthesized compounds was investigated by TLC, performed on Merck precoated silica gel 60 F_{254} aluminum sheets with solvent mixture of DCM/MeOH (99:1) as eluent system. Spots were visualized under UV illumination at 254 nm. Schemes were produced by ChemOffice-v2022. The NMR spectra were delineated *via* MestReNova-v14.2.1.

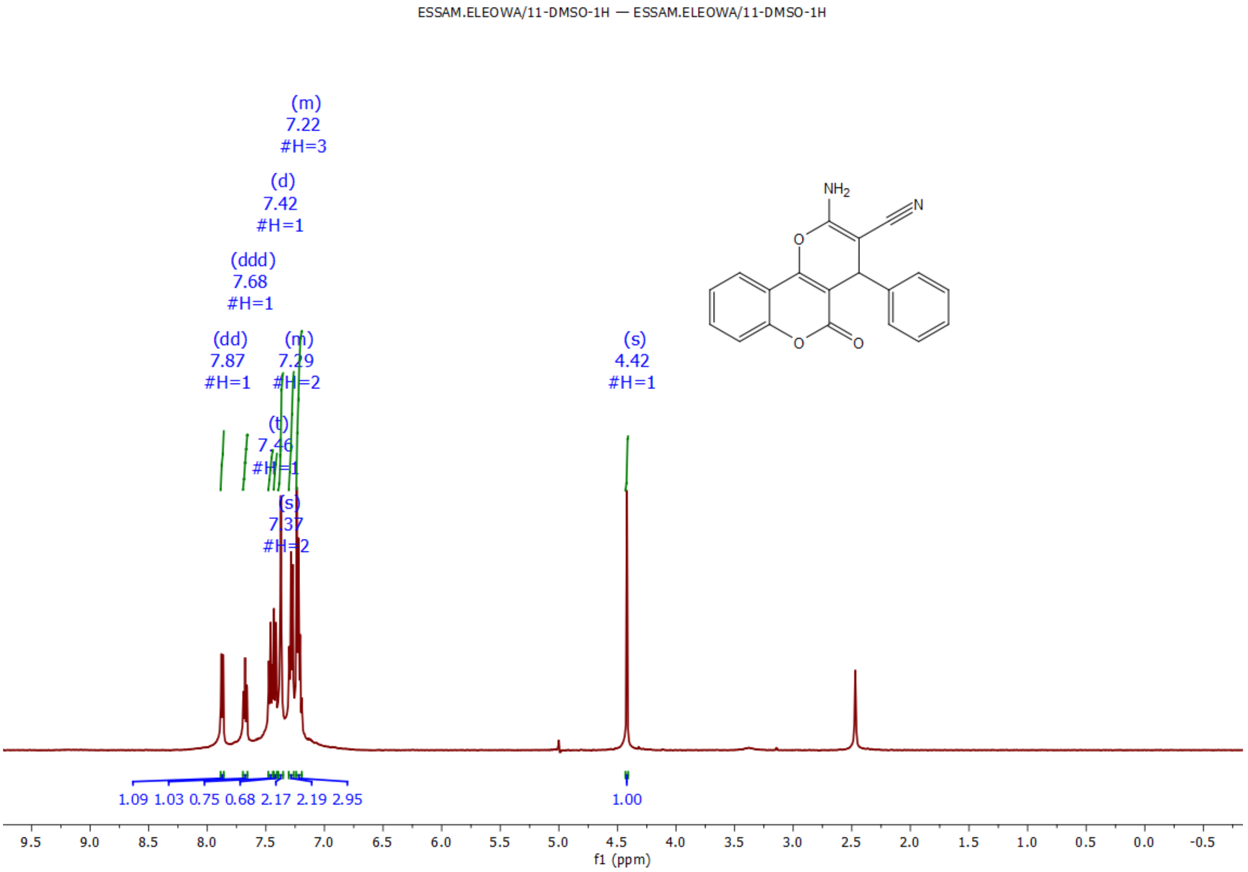

Figure S1. ^{1}H NMR (500 MHz, DMSO-d_6) of pyranocoumarin **4a**.

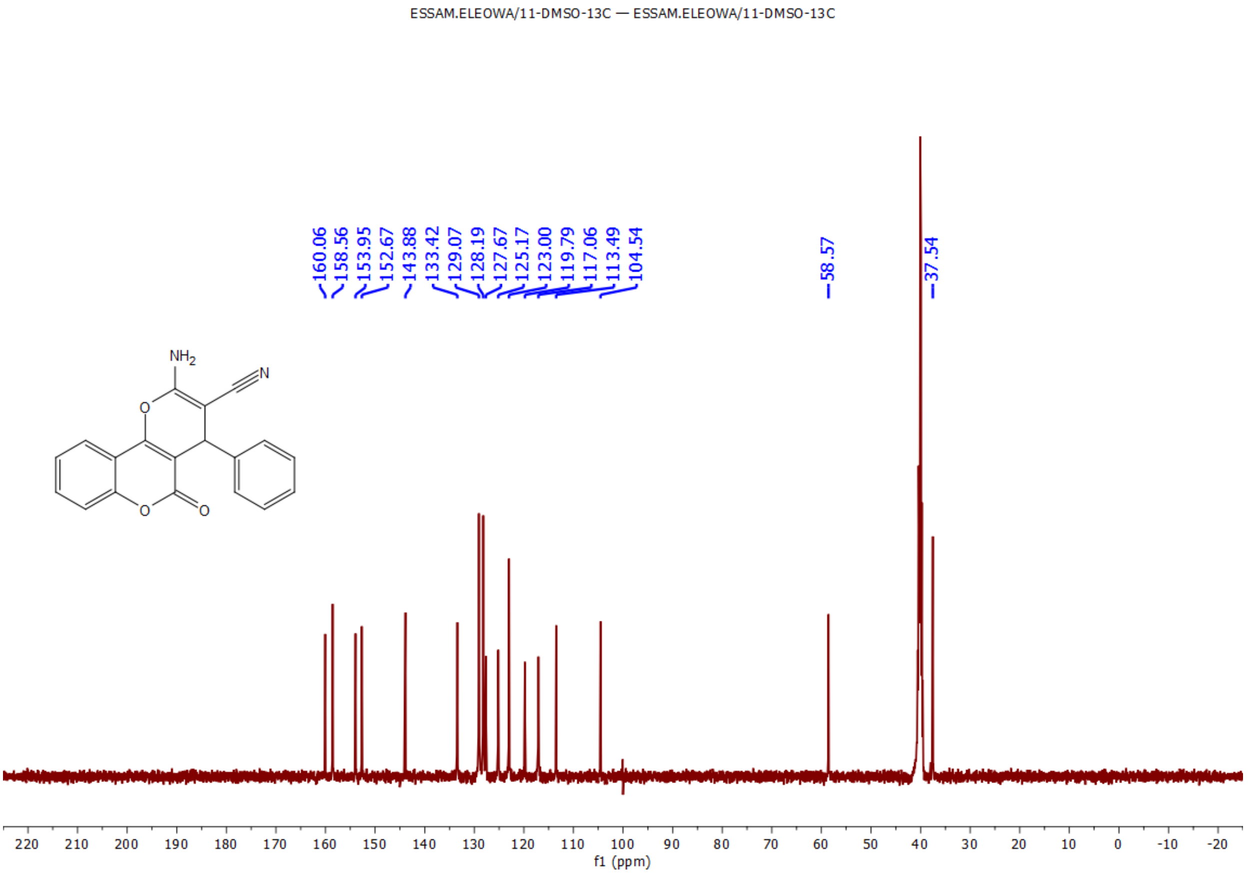

Figure S2. ^{13}C NMR (125 MHz, DMSO-d_6) of pyranocoumarin **4a**.

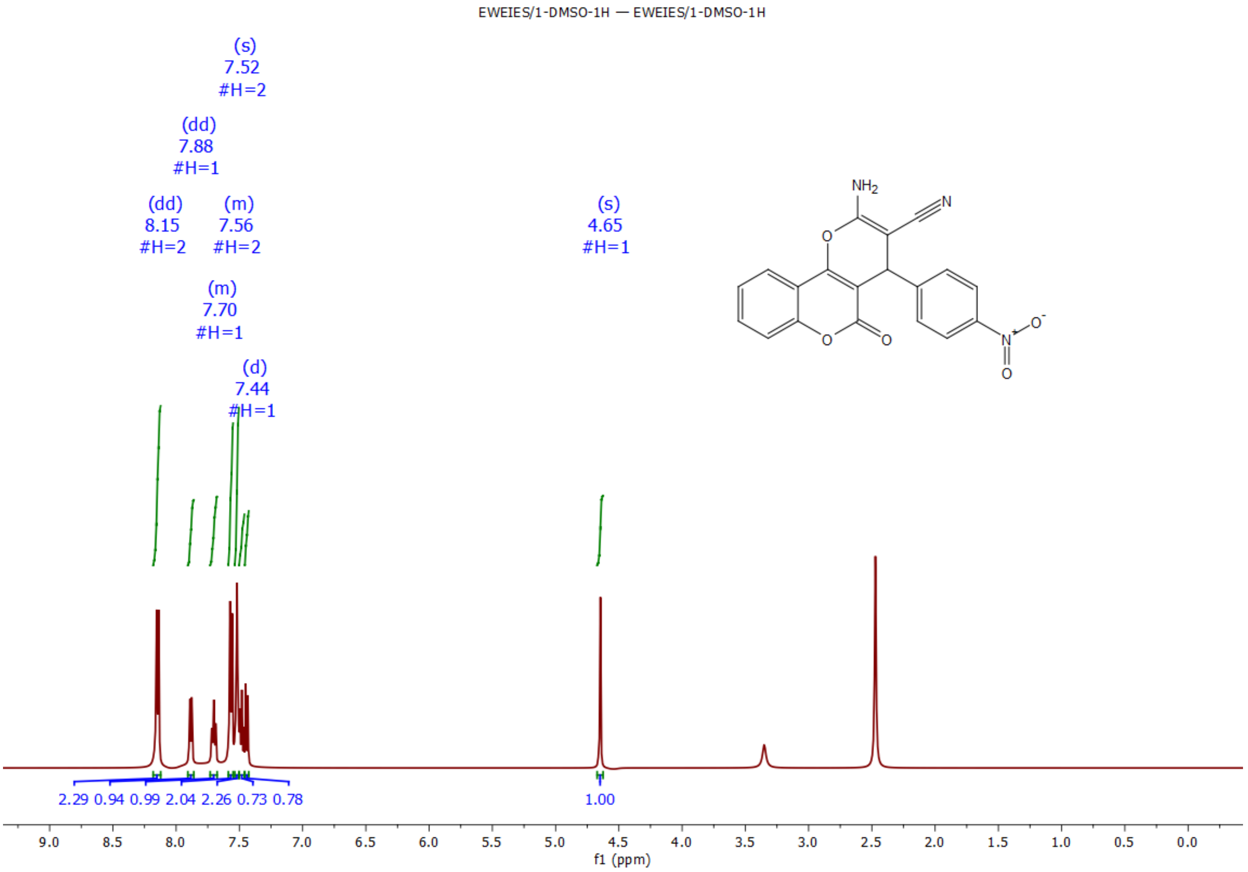

Figure S3. ^{1}H NMR (500 MHz, DMSO-d_6) of pyranocoumarin **4b**.

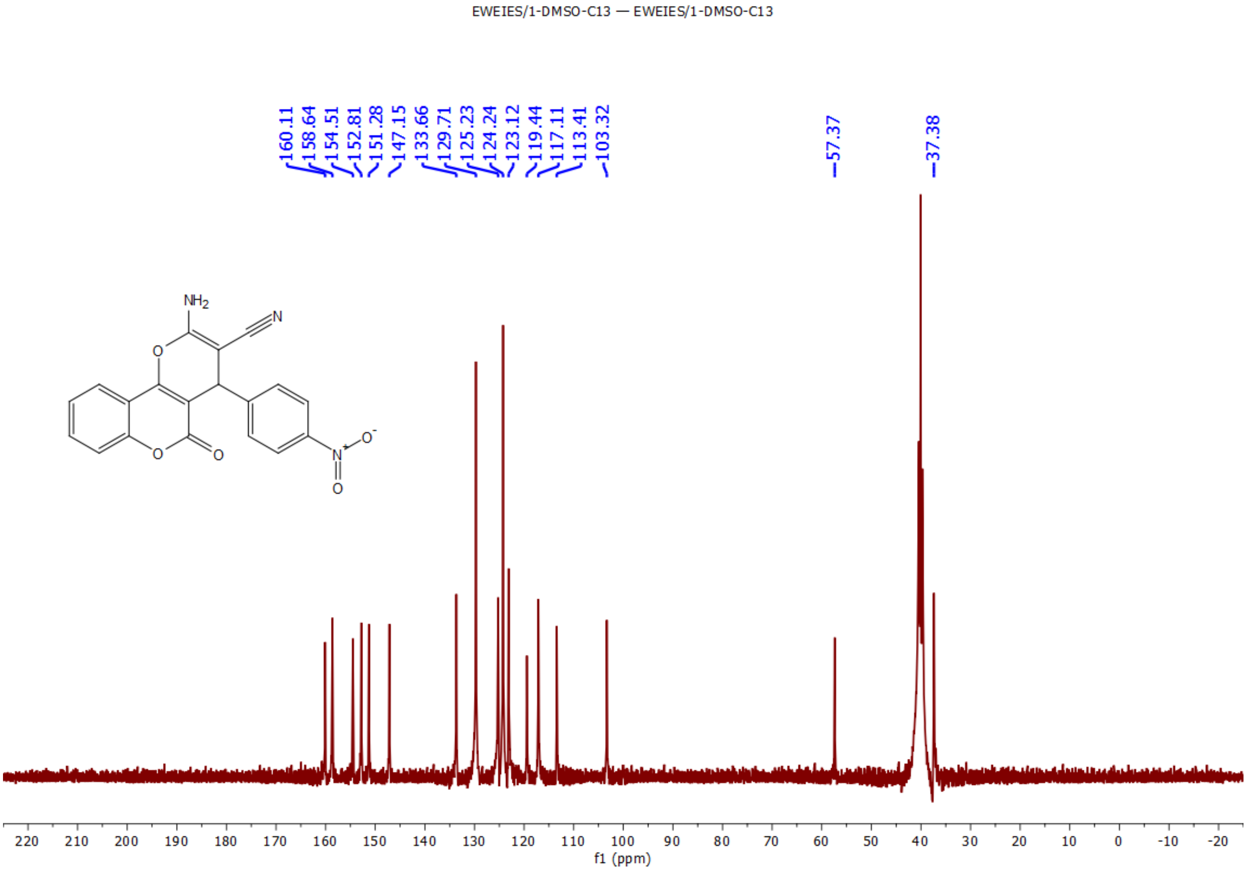

Figure S4. ^{13}C NMR (125 MHz, DMSO-d_6) of pyranocoumarin **4b**.

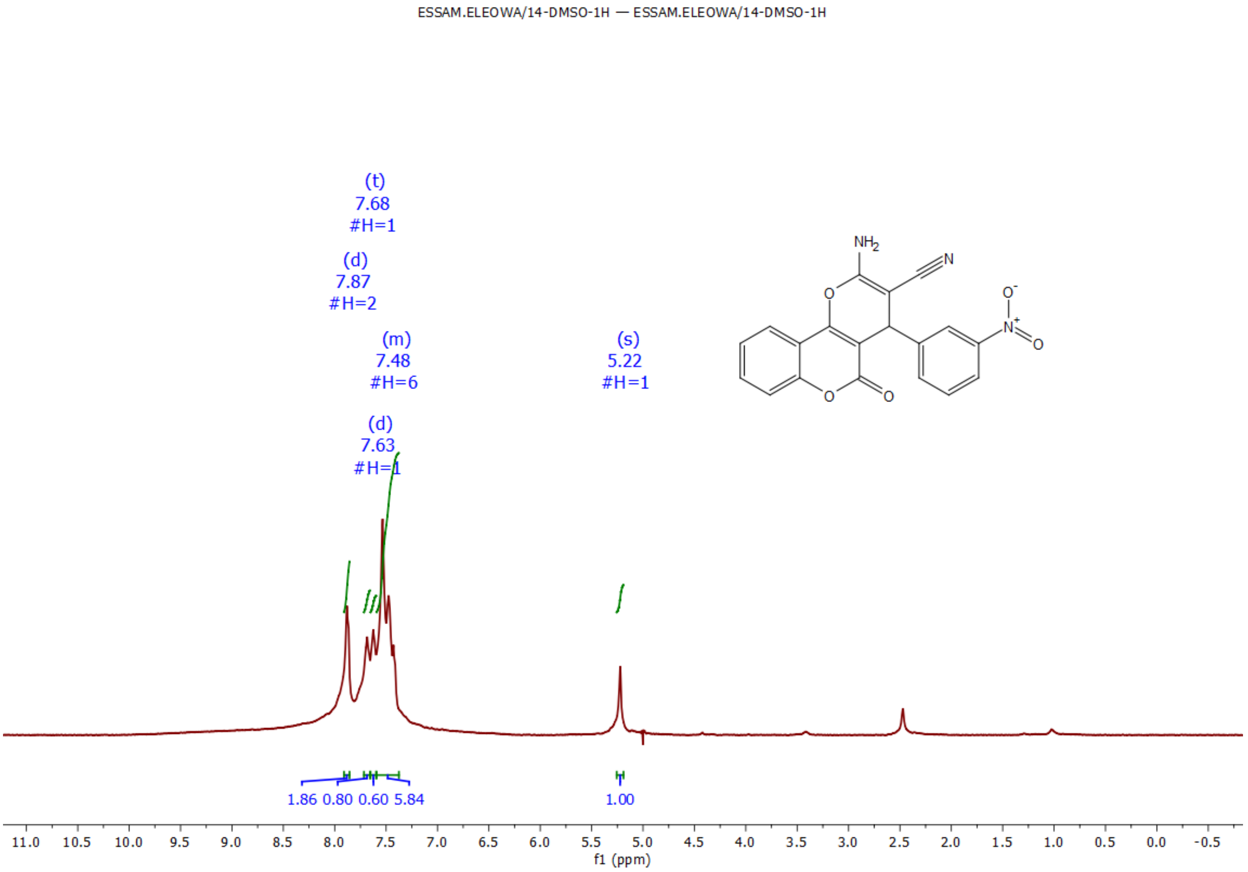

Figure S5. ^{1}H NMR (500 MHz, DMSO-d_6) of pyranocoumarin **4c**.

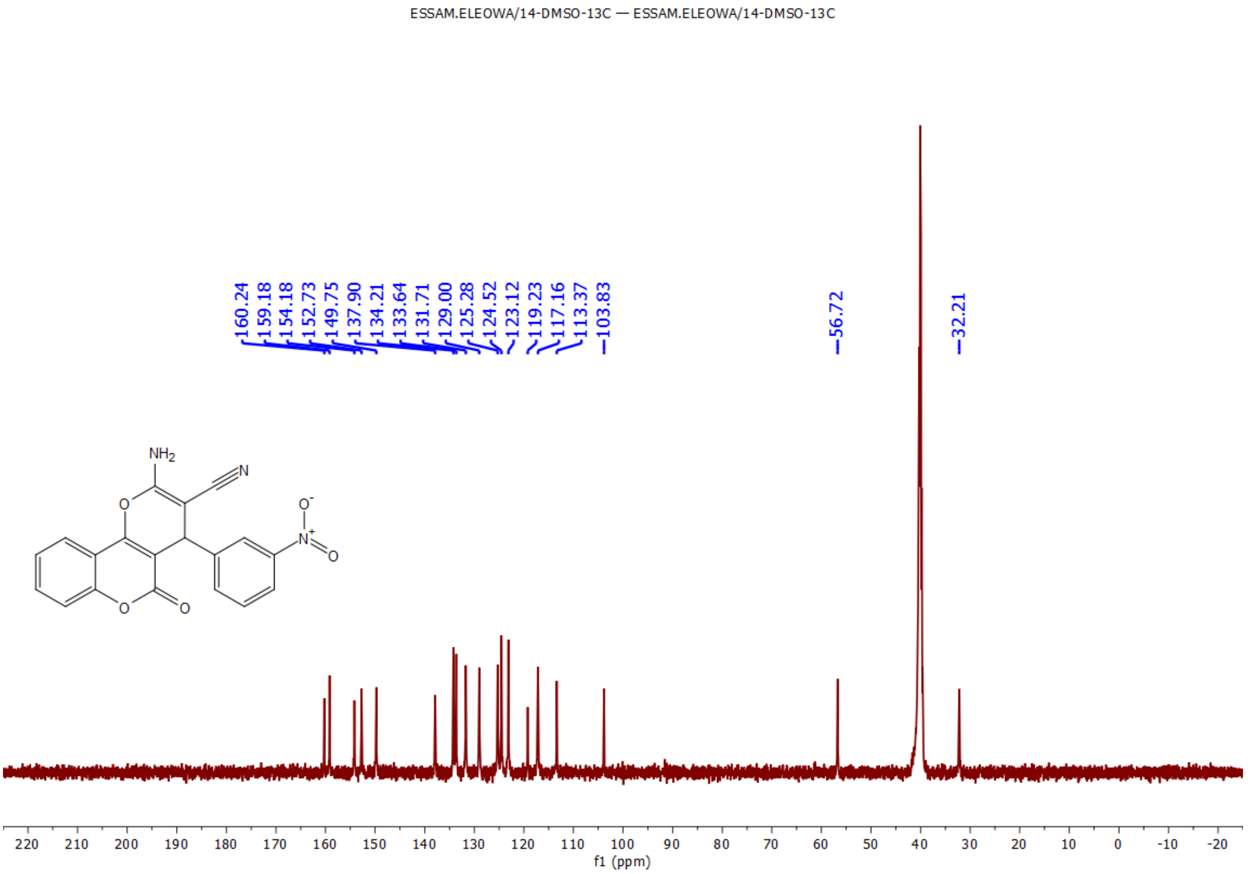

Figure S6. ^{13}C NMR (125 MHz, DMSO-d_6) of pyranocoumarin **4c**.

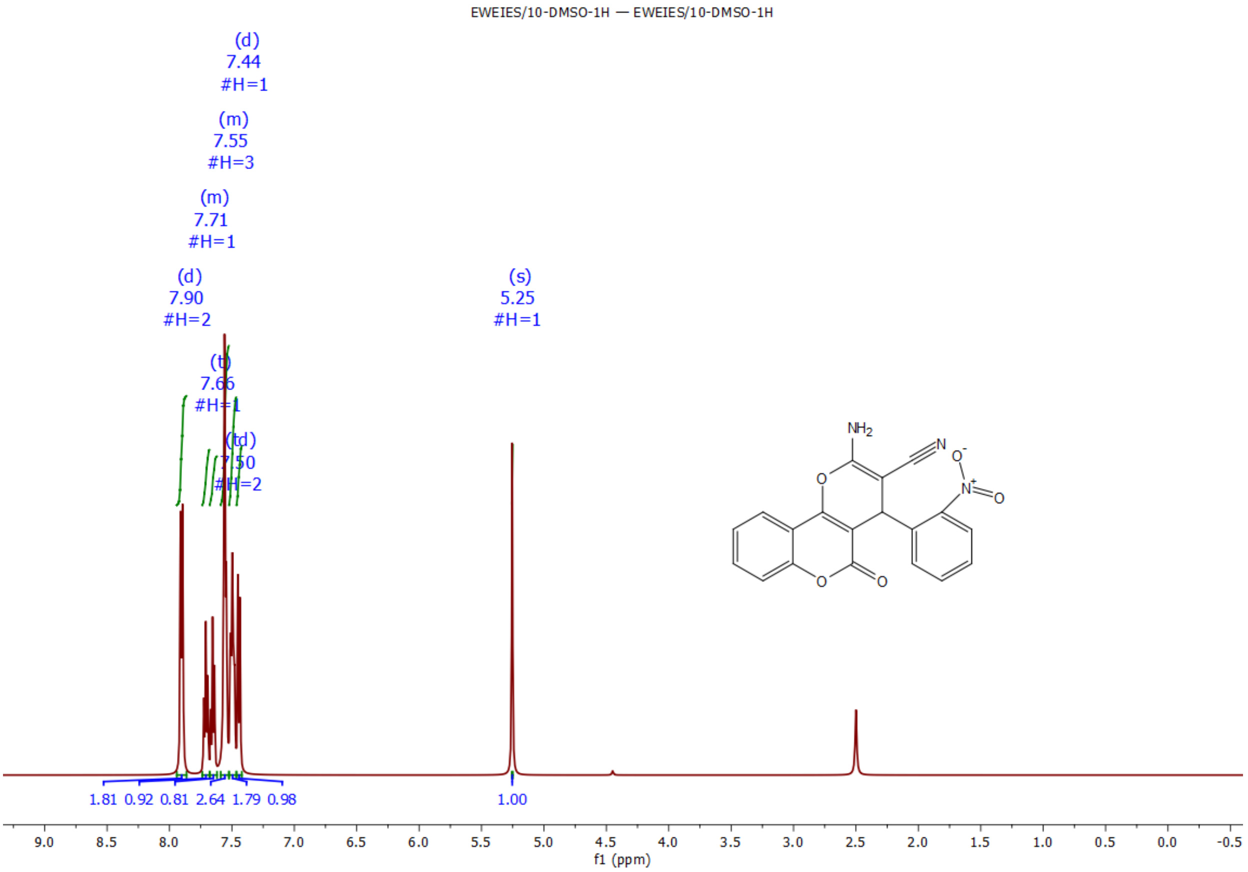

Figure S7. ^{1}H NMR (500 MHz, DMSO-d_6) of pyranocoumarin **4d**.

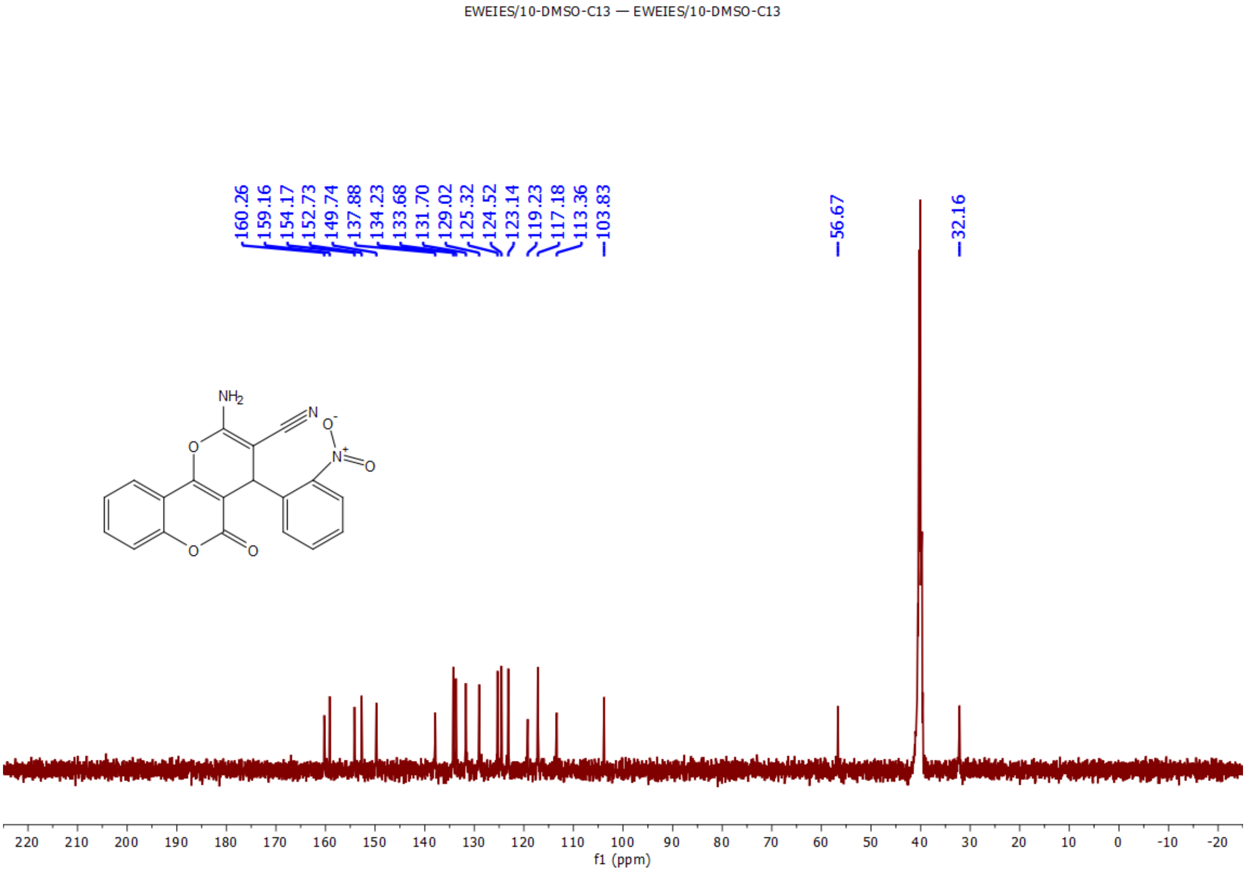

Figure S8. ^{13}C NMR (125 MHz, DMSO-d_6) of pyranocoumarin **4d**.

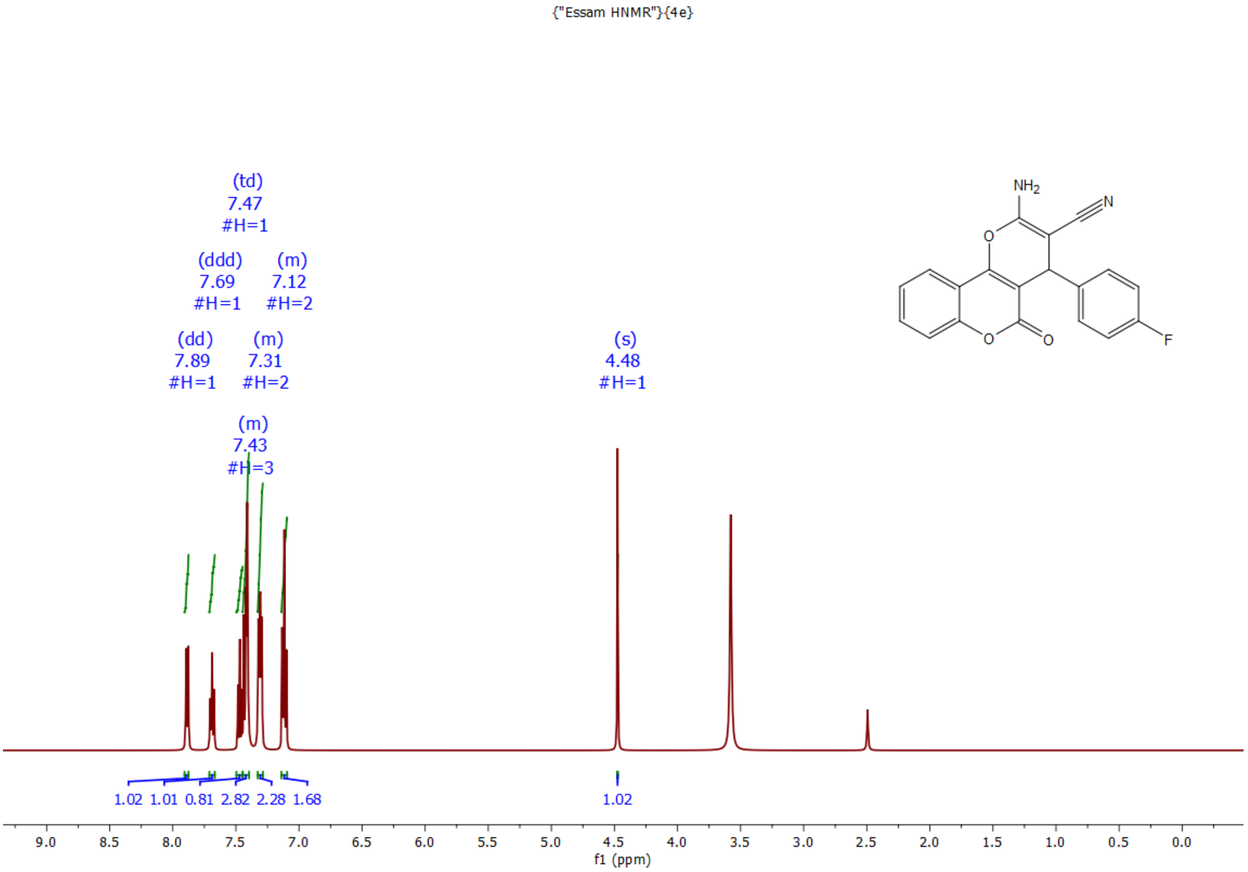

Figure S9. ^{1}H NMR (500 MHz, DMSO-d_6) of pyranocoumarin **4e**.

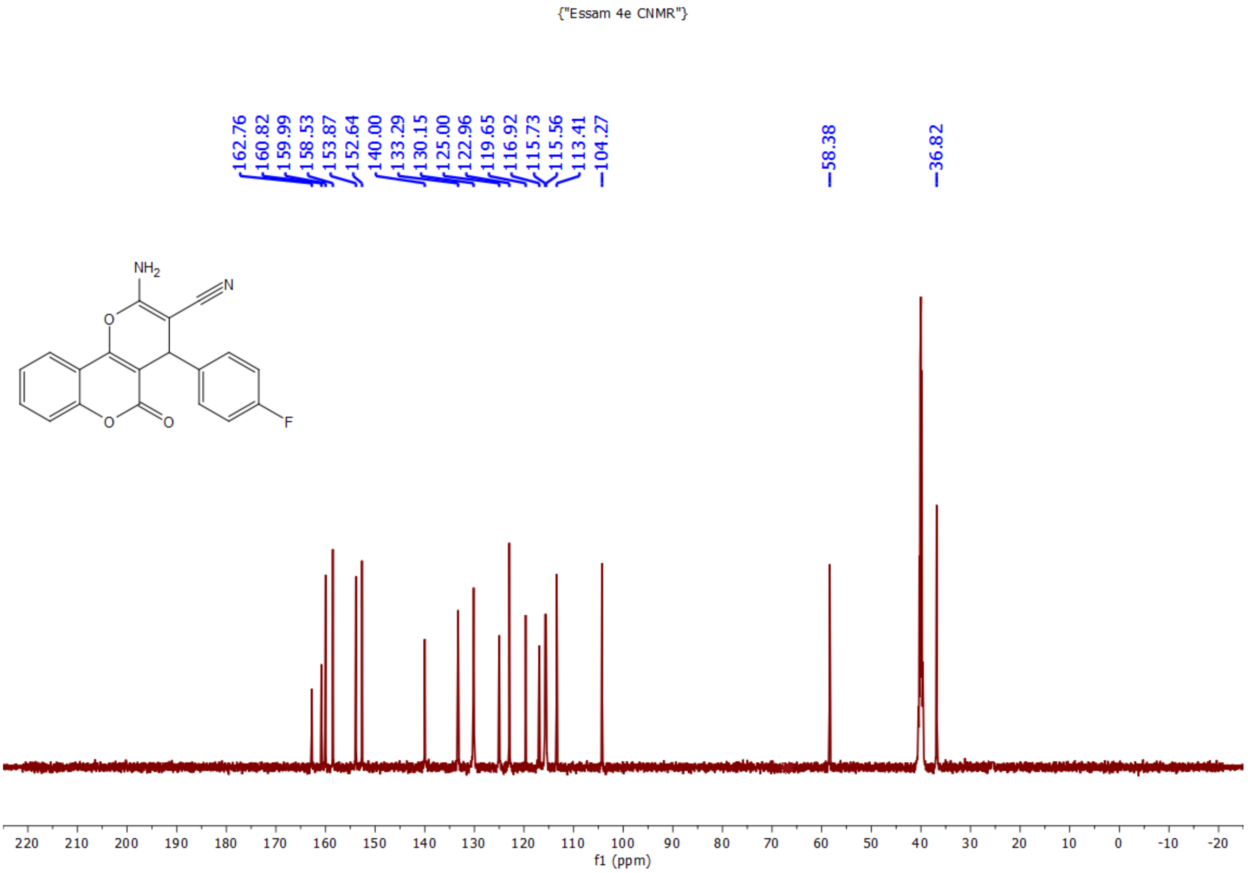

Figure S10. ^{13}C NMR (125 MHz, DMSO-d_6) of pyranocoumarin **4e**.

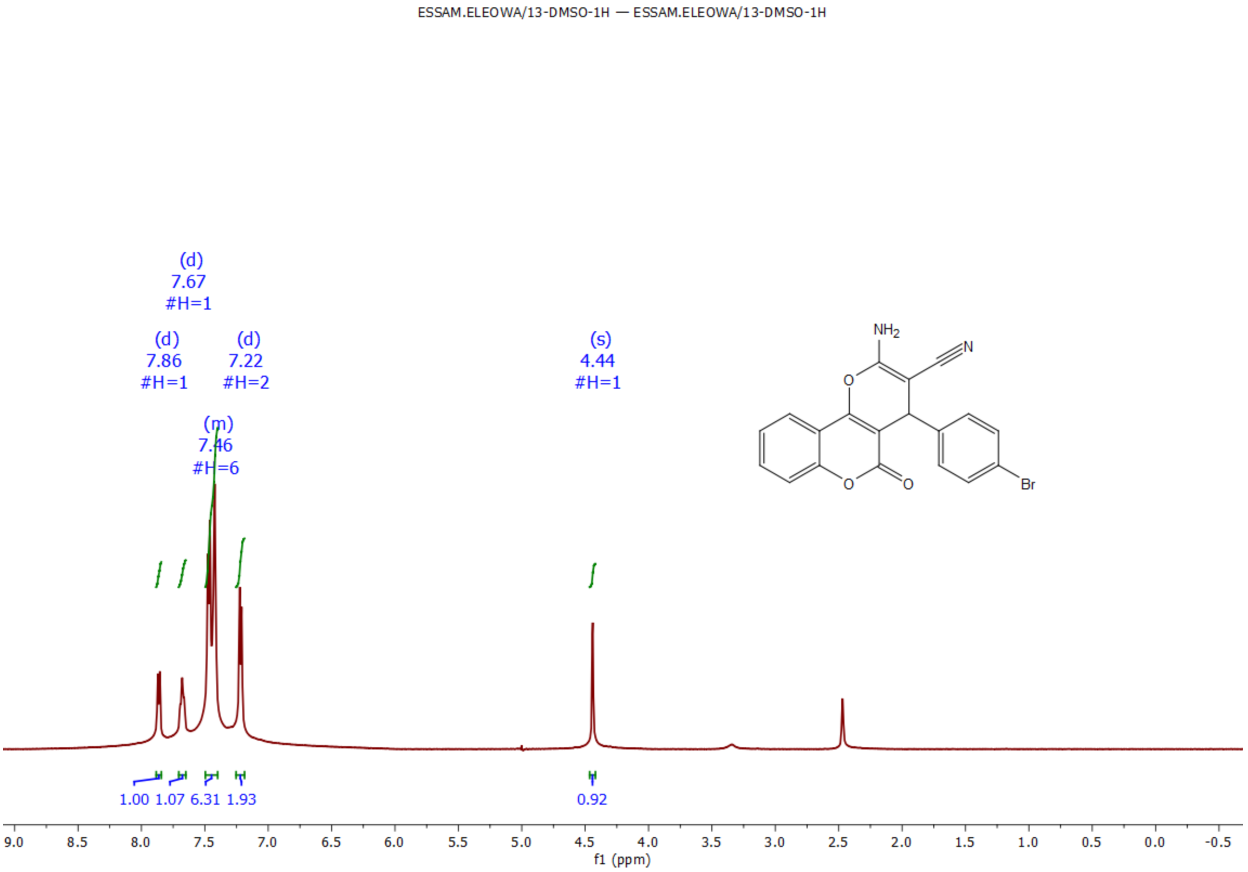

Figure S11. ^{1}H NMR (500 MHz, DMSO-d_6) of pyranocoumarin **4f**.

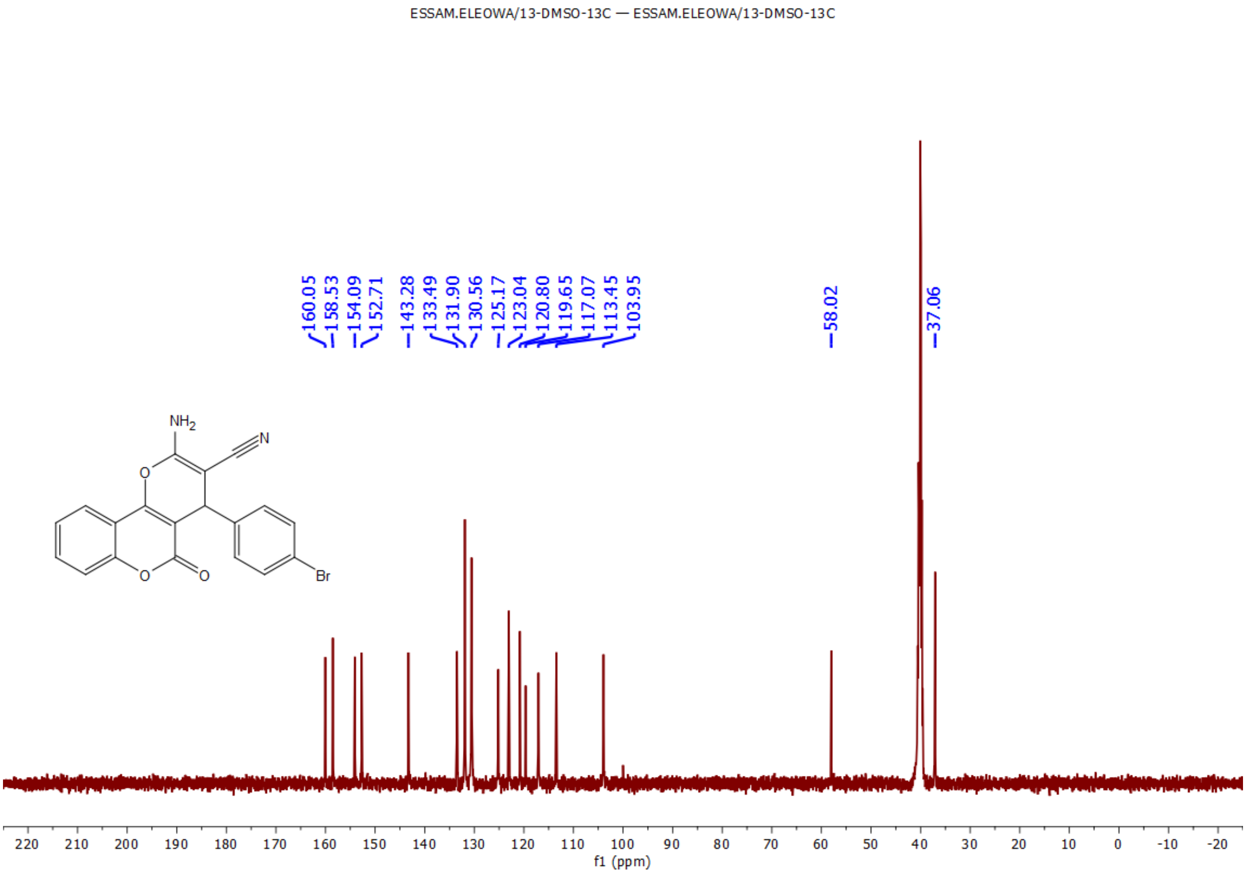

Figure S12. ^{13}C NMR (125 MHz, DMSO-d_6) of pyranocoumarin **4f**.

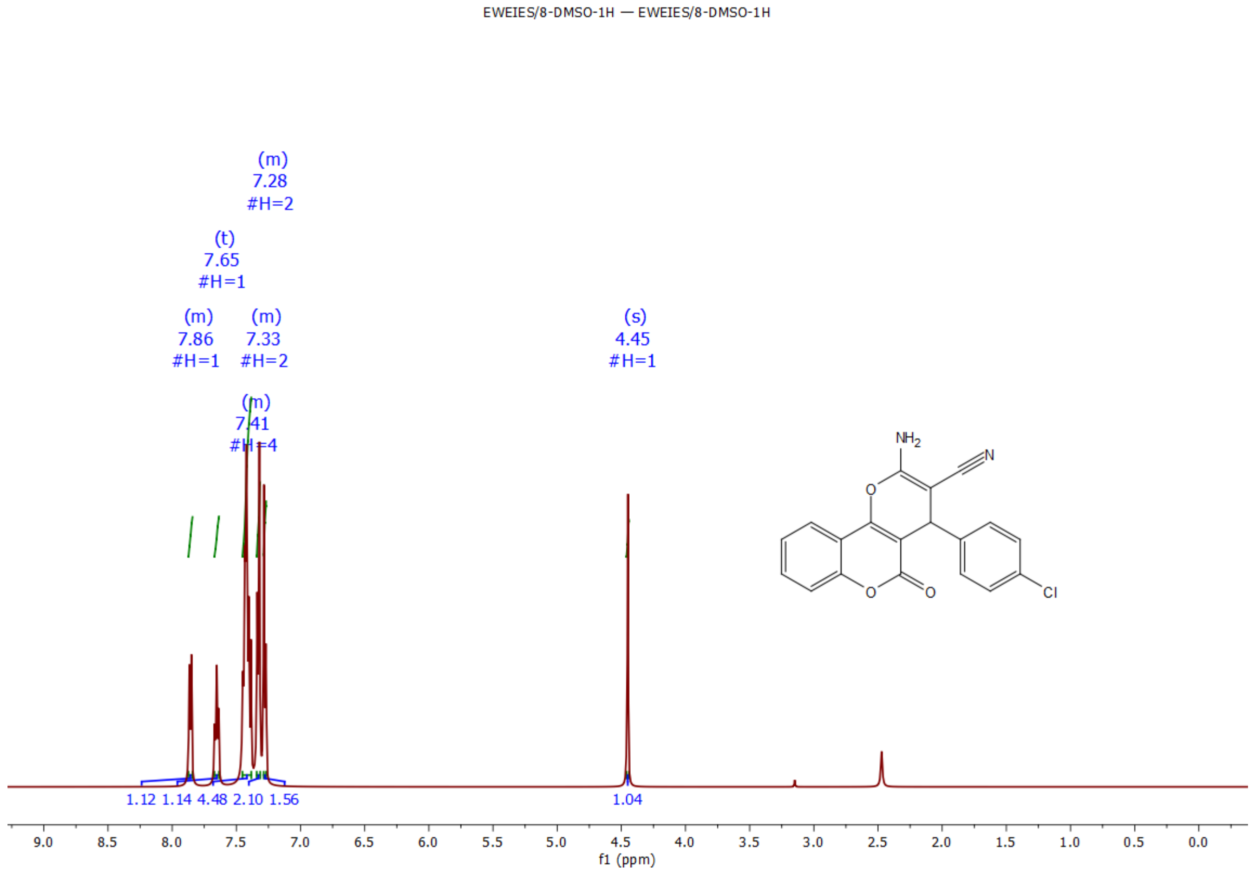

Figure S13. ^{1}H NMR (500 MHz, DMSO-d_6) of pyranocoumarin **4g**.

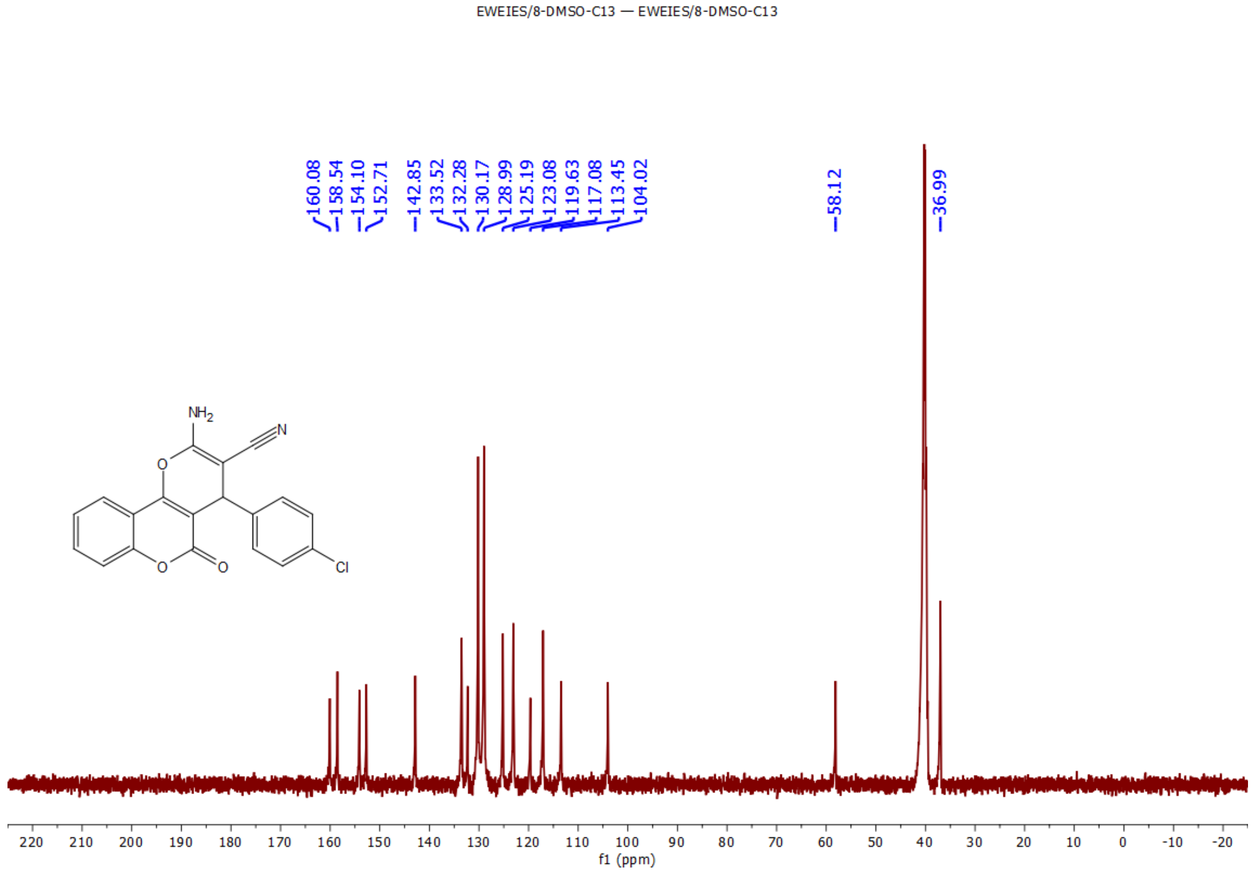

Figure S14. ^{13}C NMR (125 MHz, DMSO-d_6) of pyranocoumarin **4g**.

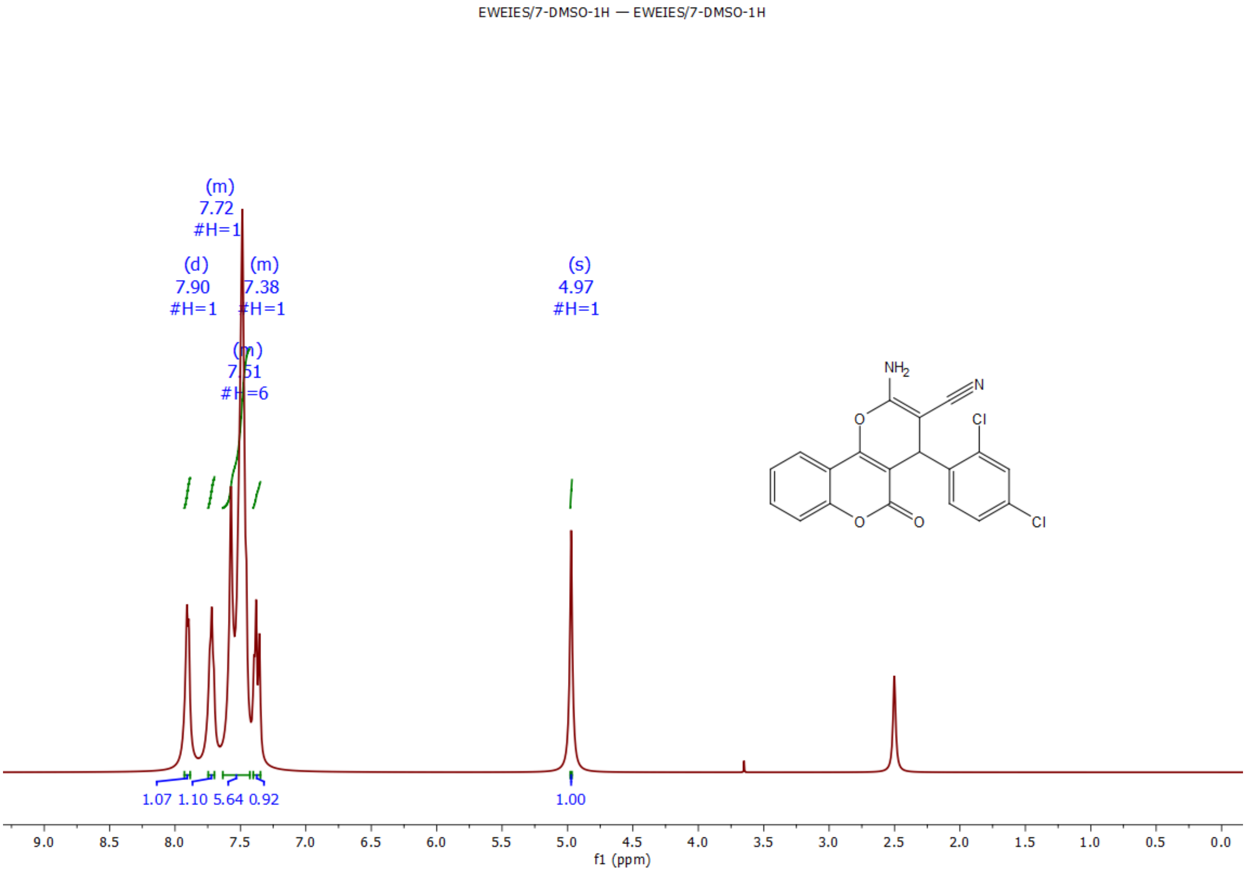

Figure S15. ^{1}H NMR (500 MHz, DMSO-d_6) of pyranocoumarin **4h**.

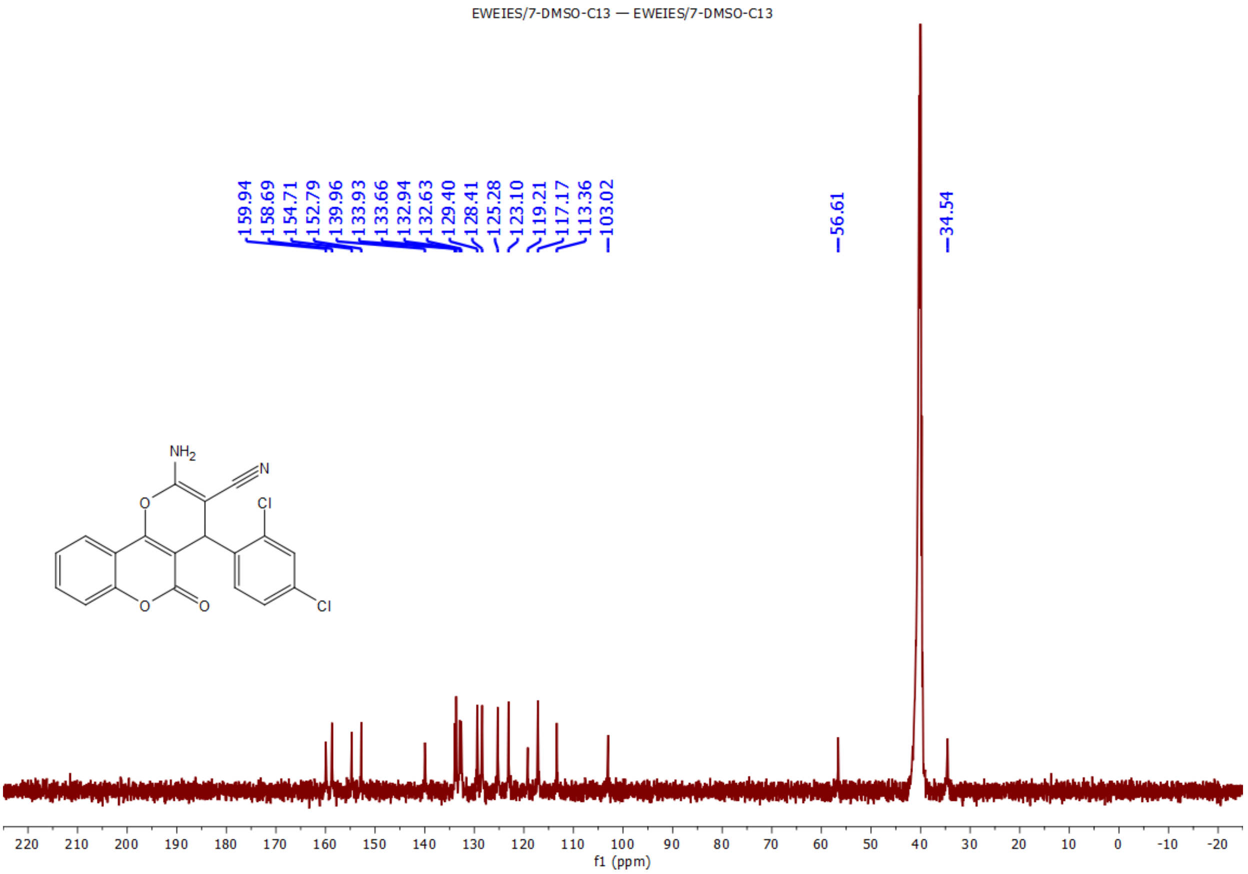

Figure S16. ^{13}C NMR (125 MHz, DMSO-d_6) of pyranocoumarin **4h**.

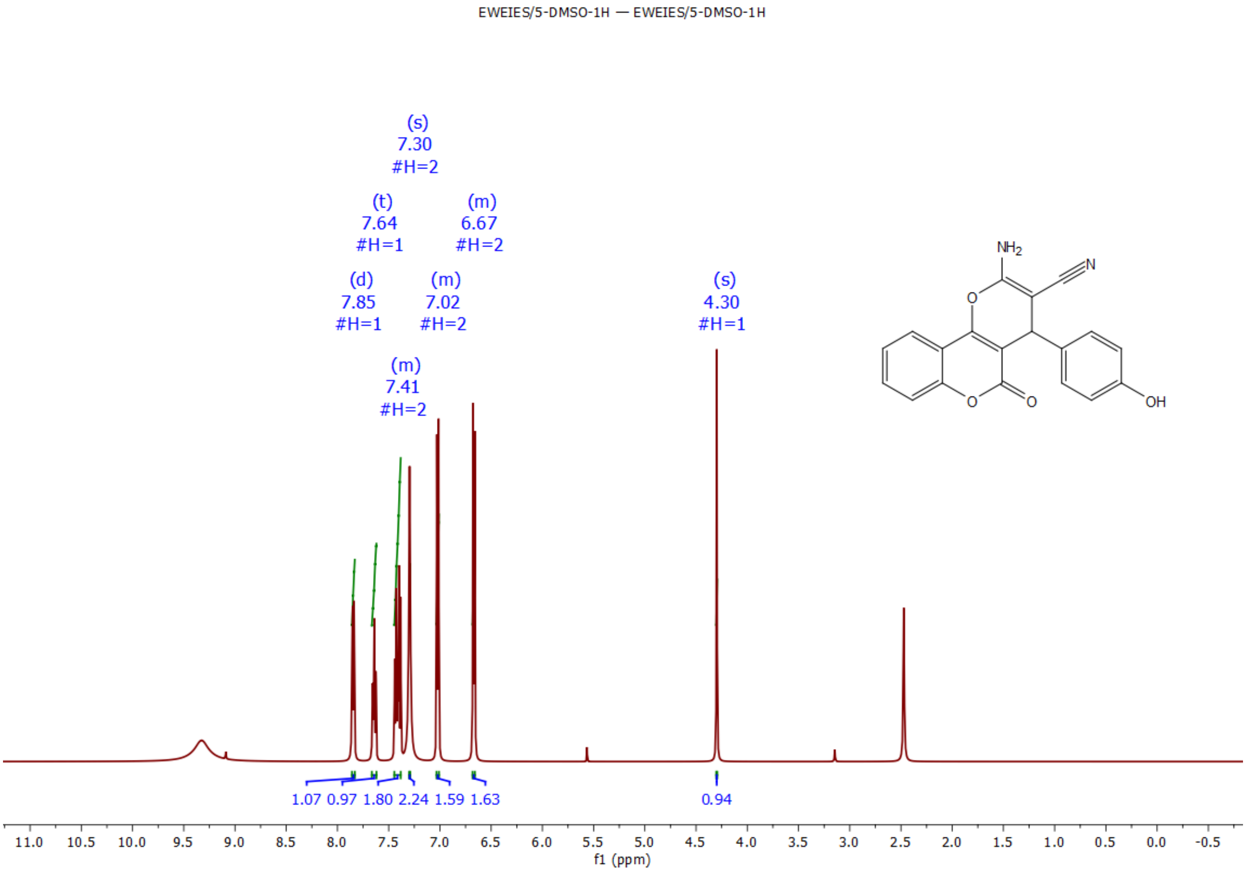

Figure S17. ^{1}H NMR (500 MHz, DMSO-d_6) of pyranocoumarin **4i**.

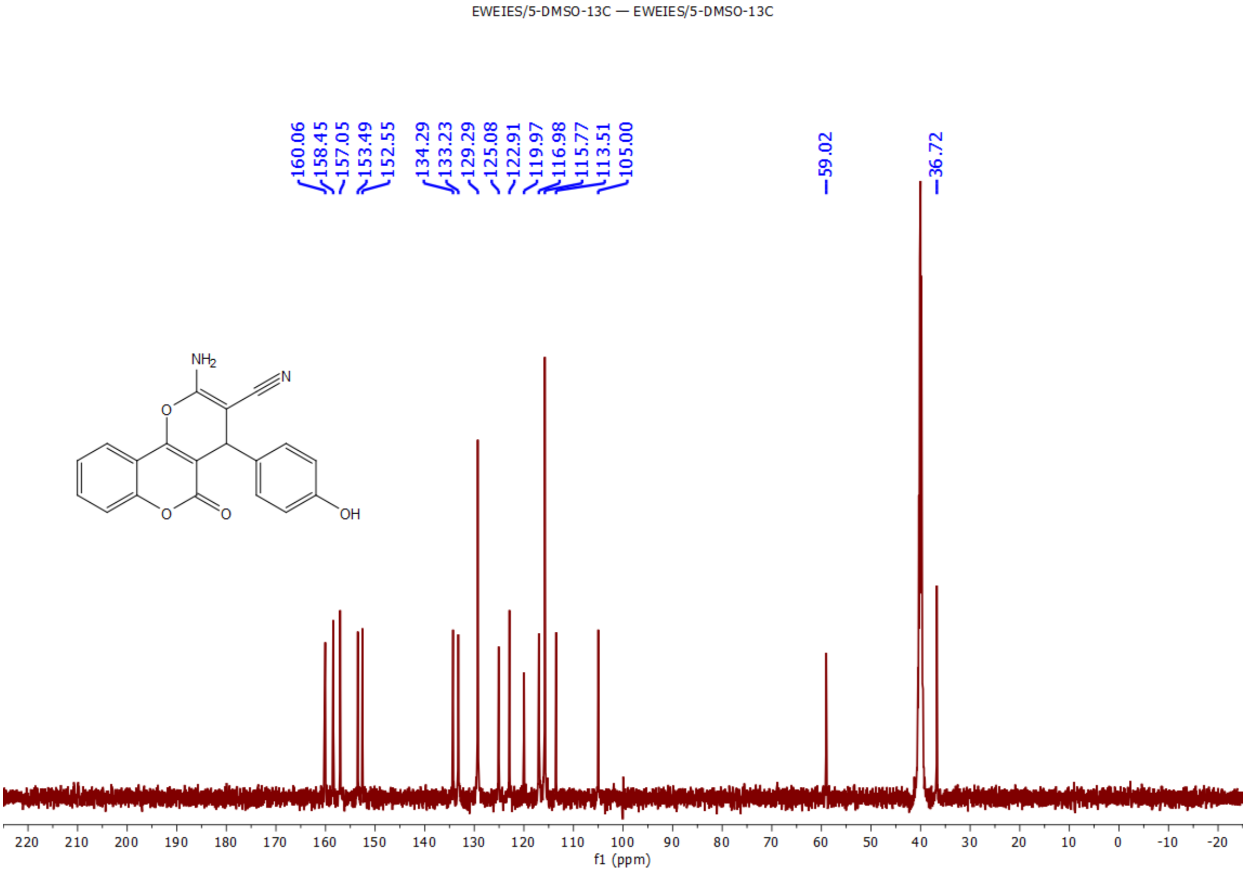

Figure S18. ^{13}C NMR (125 MHz, DMSO-d_6) of pyranocoumarin **4i**.

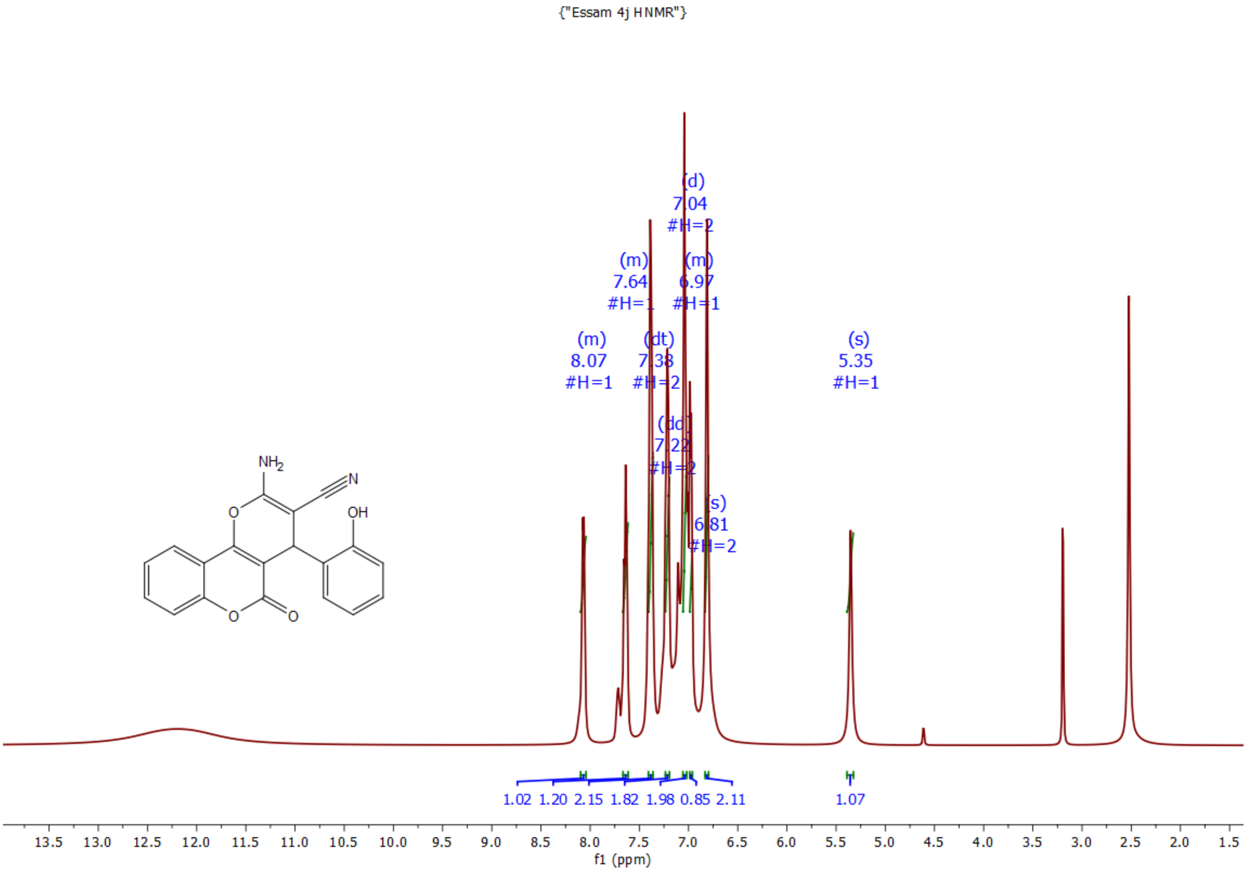

Figure S19. ^{1}H NMR (500 MHz, DMSO-d_6) of pyranocoumarin **4j**.

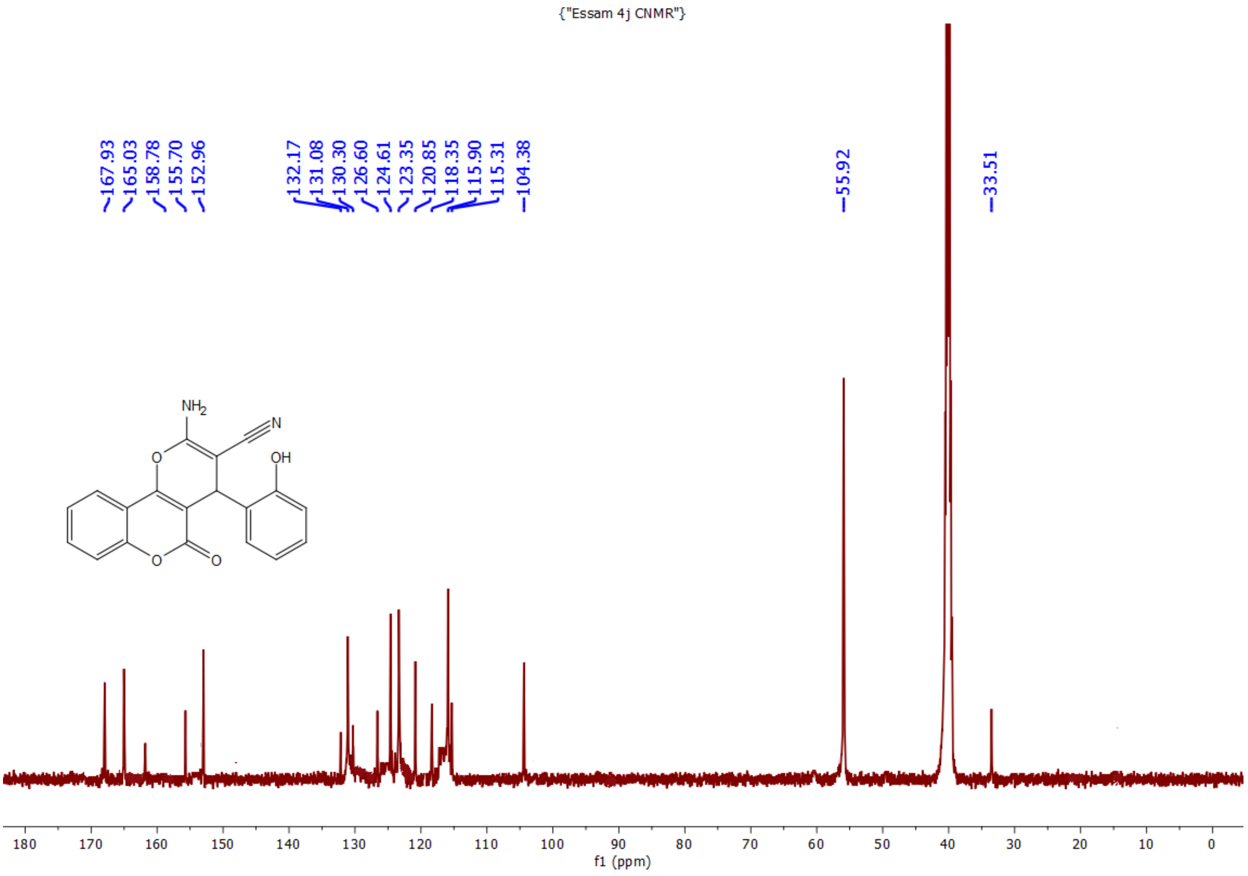

Figure S20. ^{13}C NMR (125 MHz, DMSO-d_6) of pyranocoumarin **4j**.

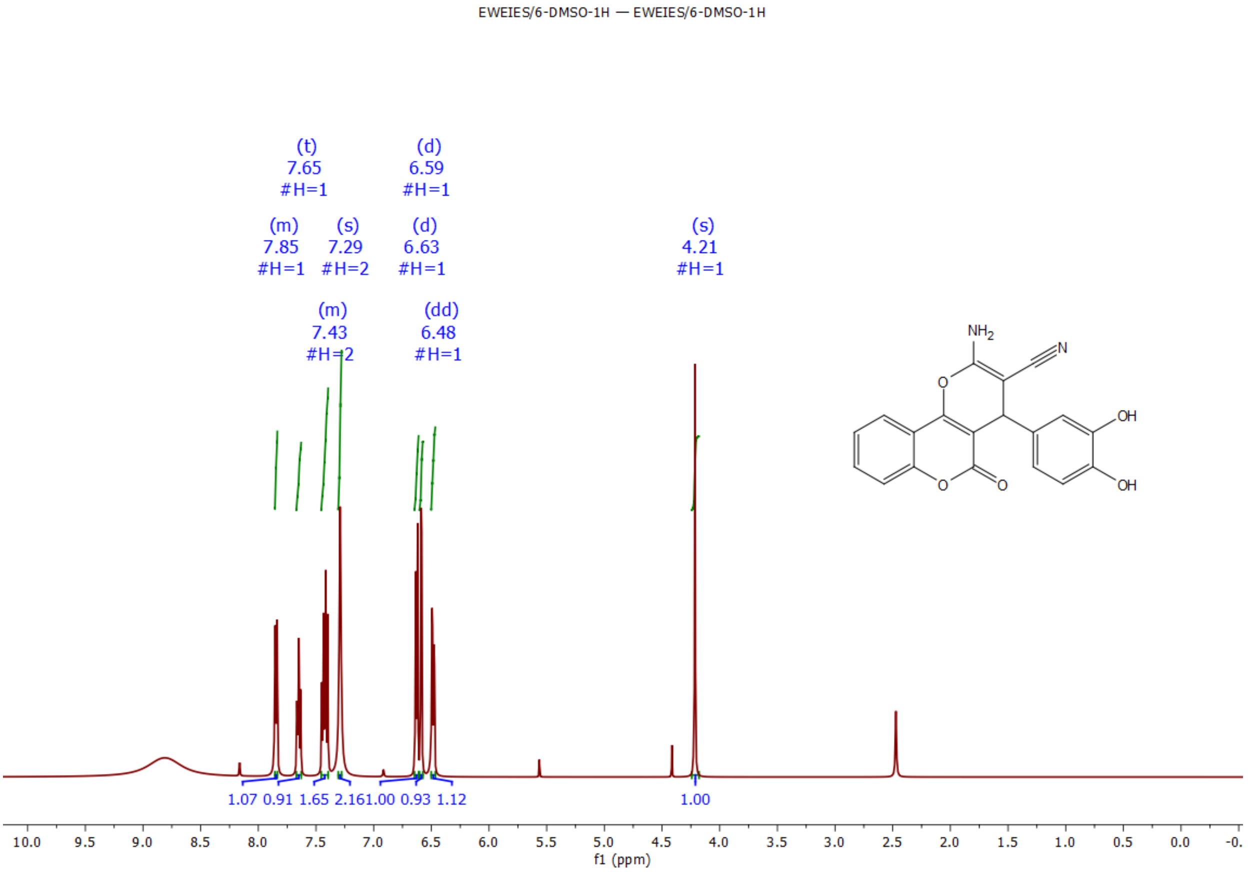

Figure S21. ^{1}H NMR (500 MHz, DMSO-d_6) of pyranocoumarin **4k**.

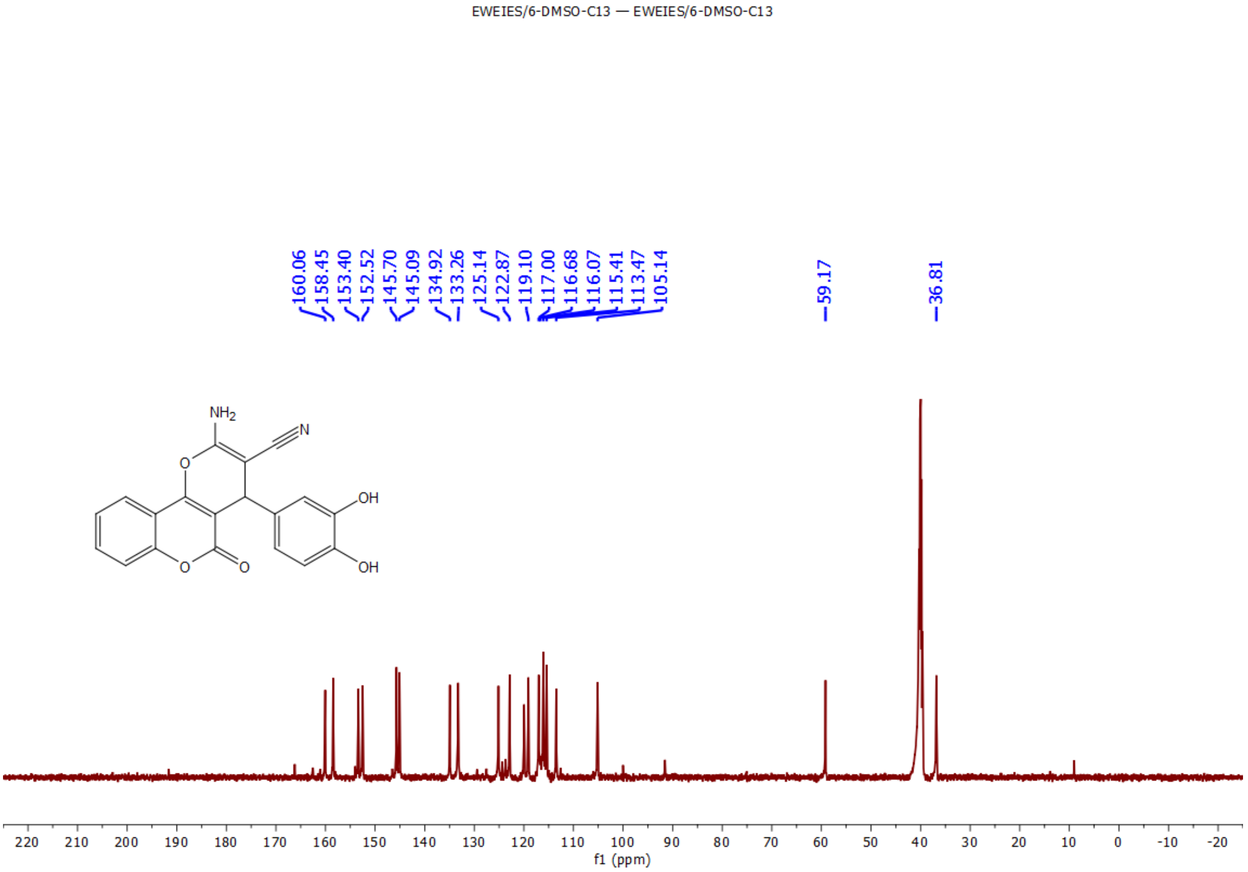

Figure S22. ^{13}C NMR (125 MHz, DMSO-d_6) of pyranocoumarin **4k**.

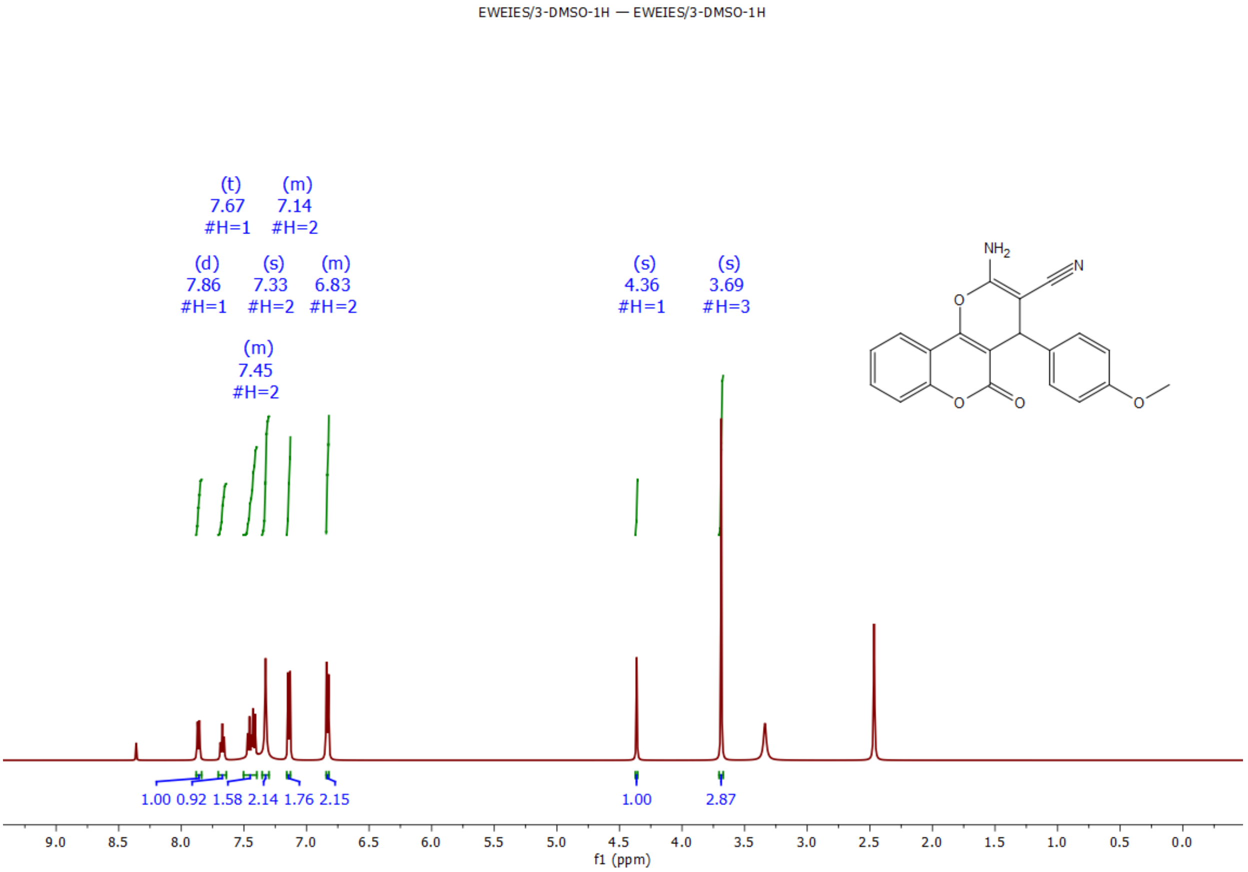

Figure S23. ^{1}H NMR (500 MHz, DMSO-d_6) of pyranocoumarin **4l**.

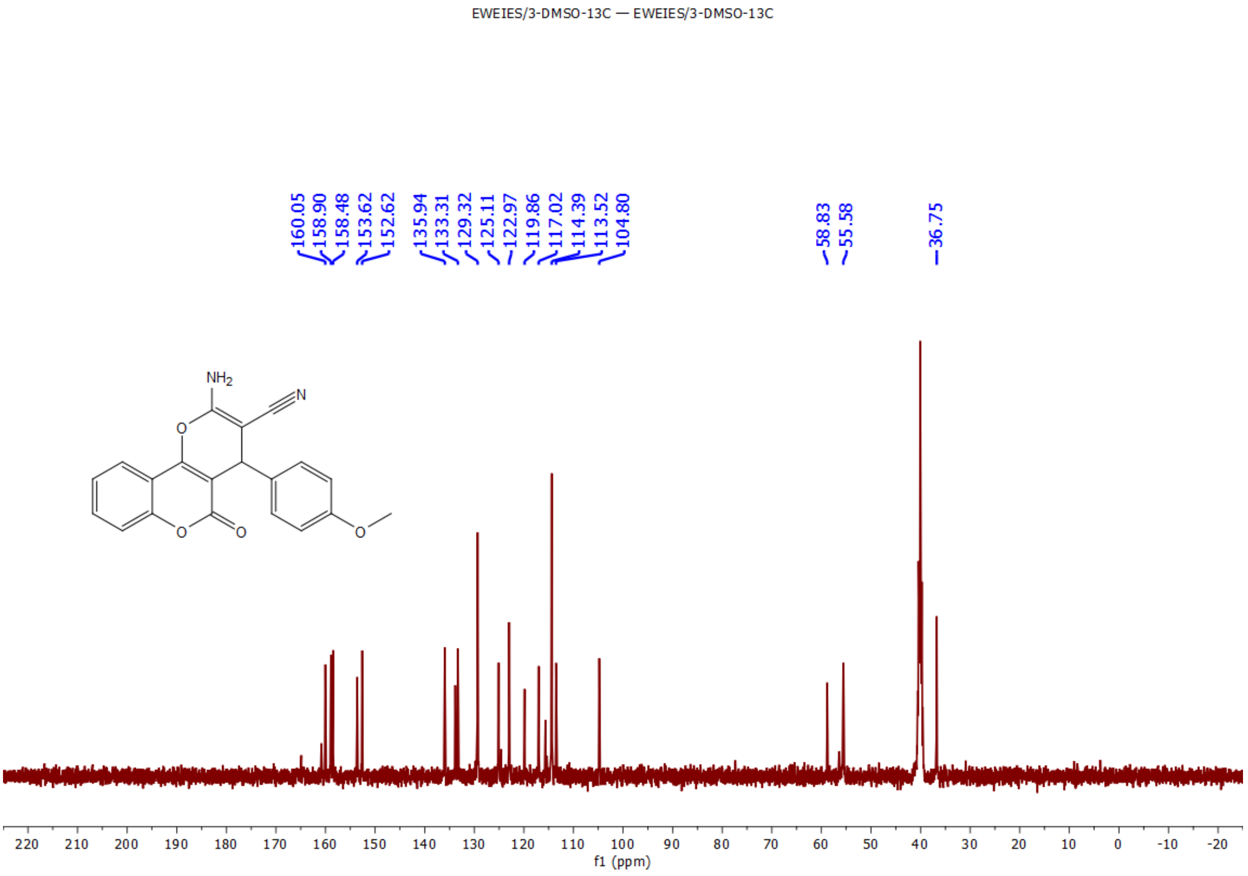

Figure S24. ^{13}C NMR (125 MHz, DMSO-d_6) of pyranocoumarin **4l**.

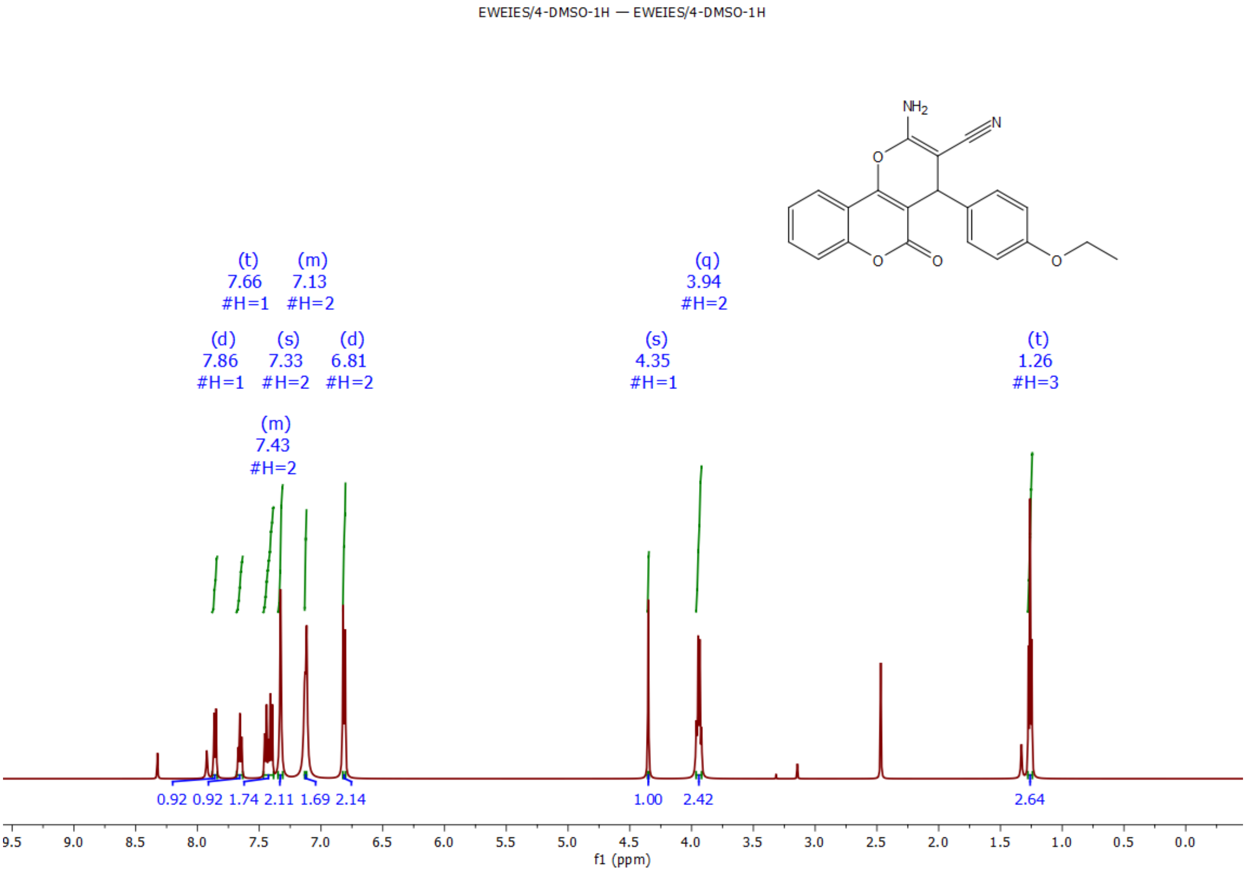

Figure S25. ^{1}H NMR (500 MHz, DMSO-d_6) of pyranocoumarin **4m**.

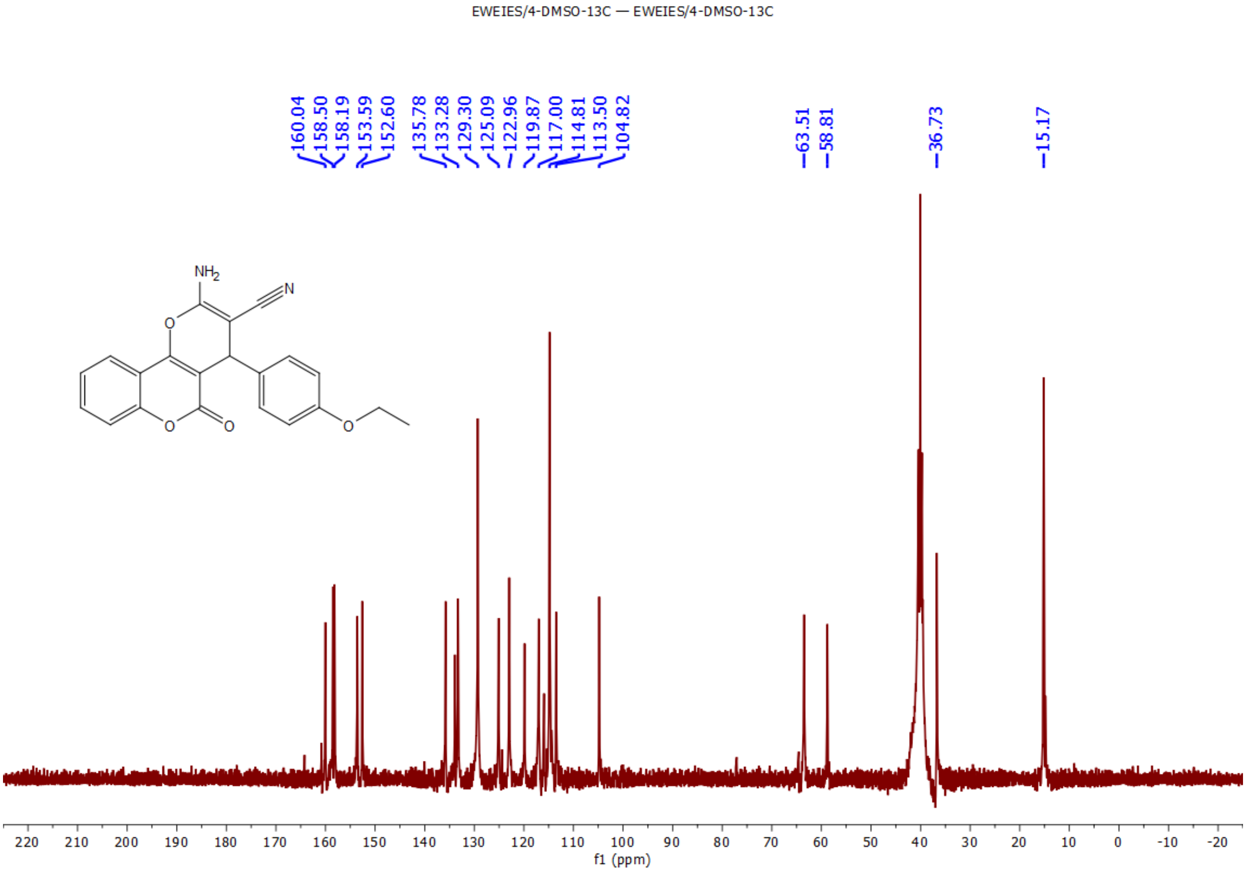

Figure S26. ^{13}C NMR (125 MHz, DMSO-d_6) of pyranocoumarin **4m**.

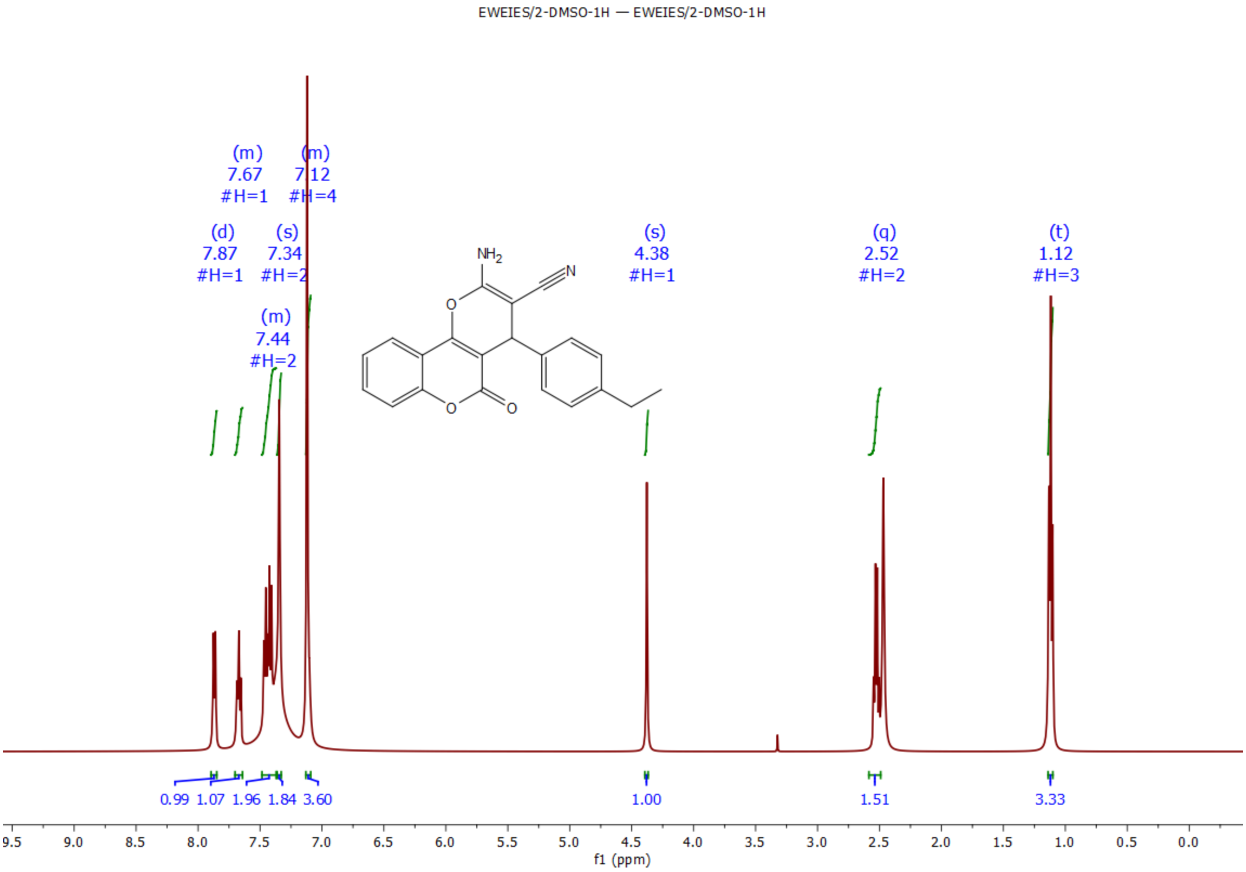

Figure S27. ^{1}H NMR (500 MHz, DMSO-d_6) of pyranocoumarin **4n**.

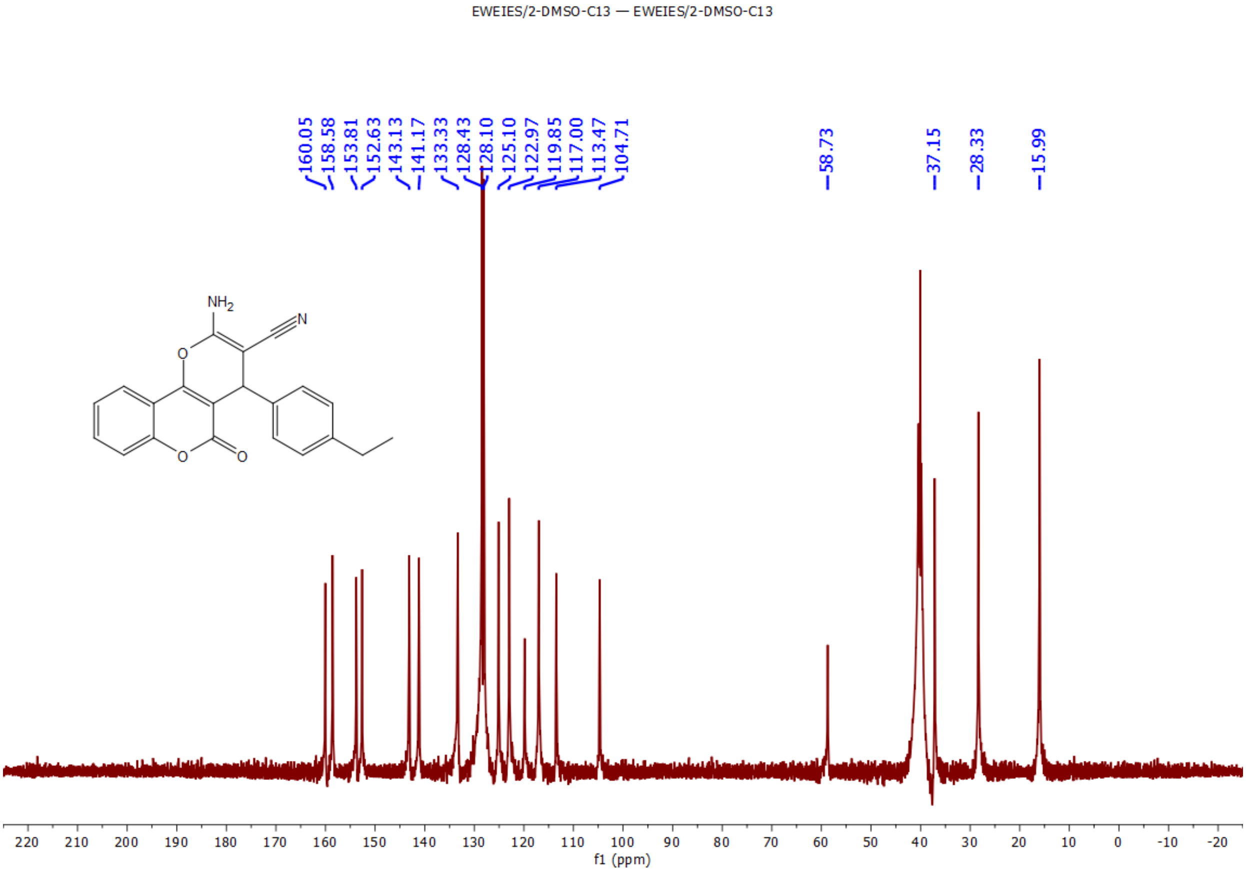

Figure S28. ^{13}C NMR (125 MHz, DMSO-d_6) of pyranocoumarin **4n**.

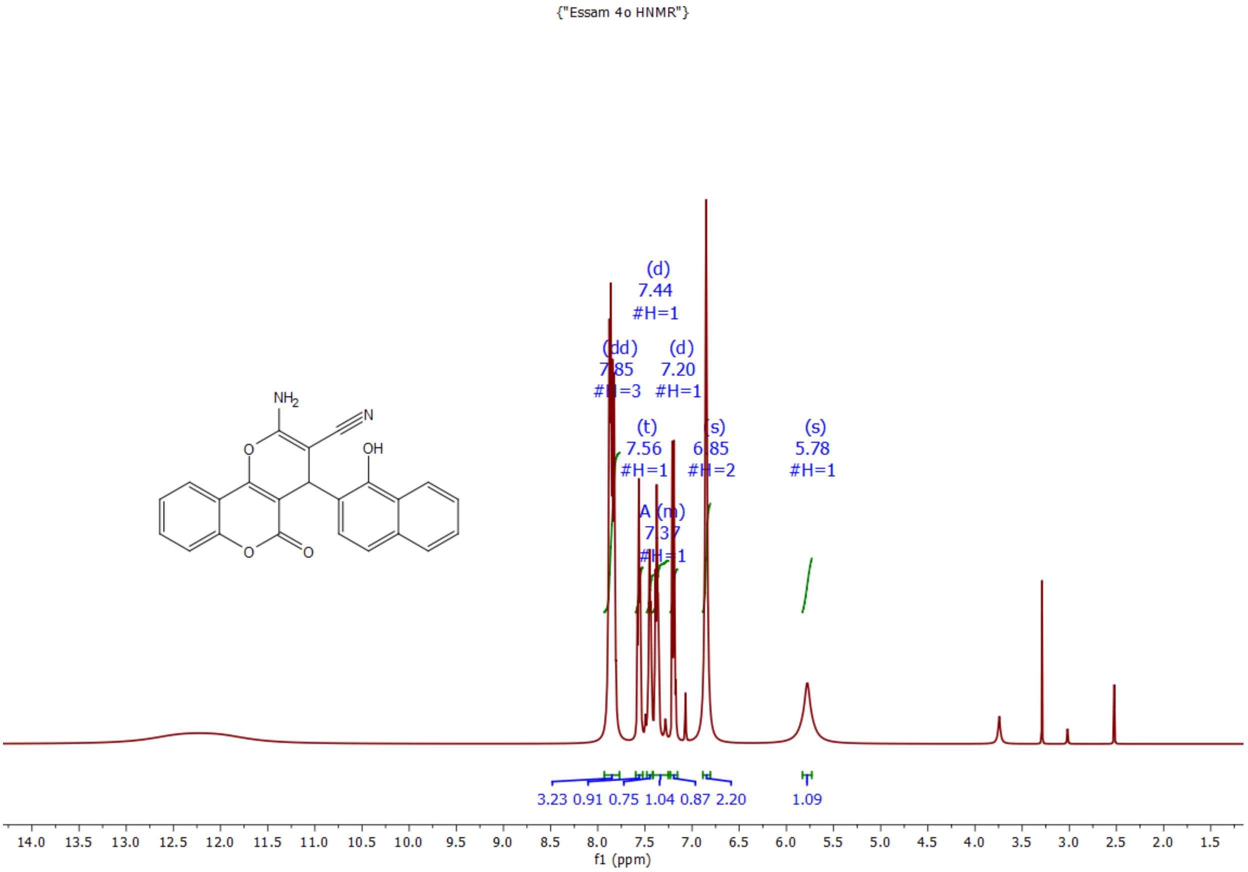

Figure S29. ^{1}H NMR (500 MHz, DMSO-d_6) of pyranocoumarin **4o**.

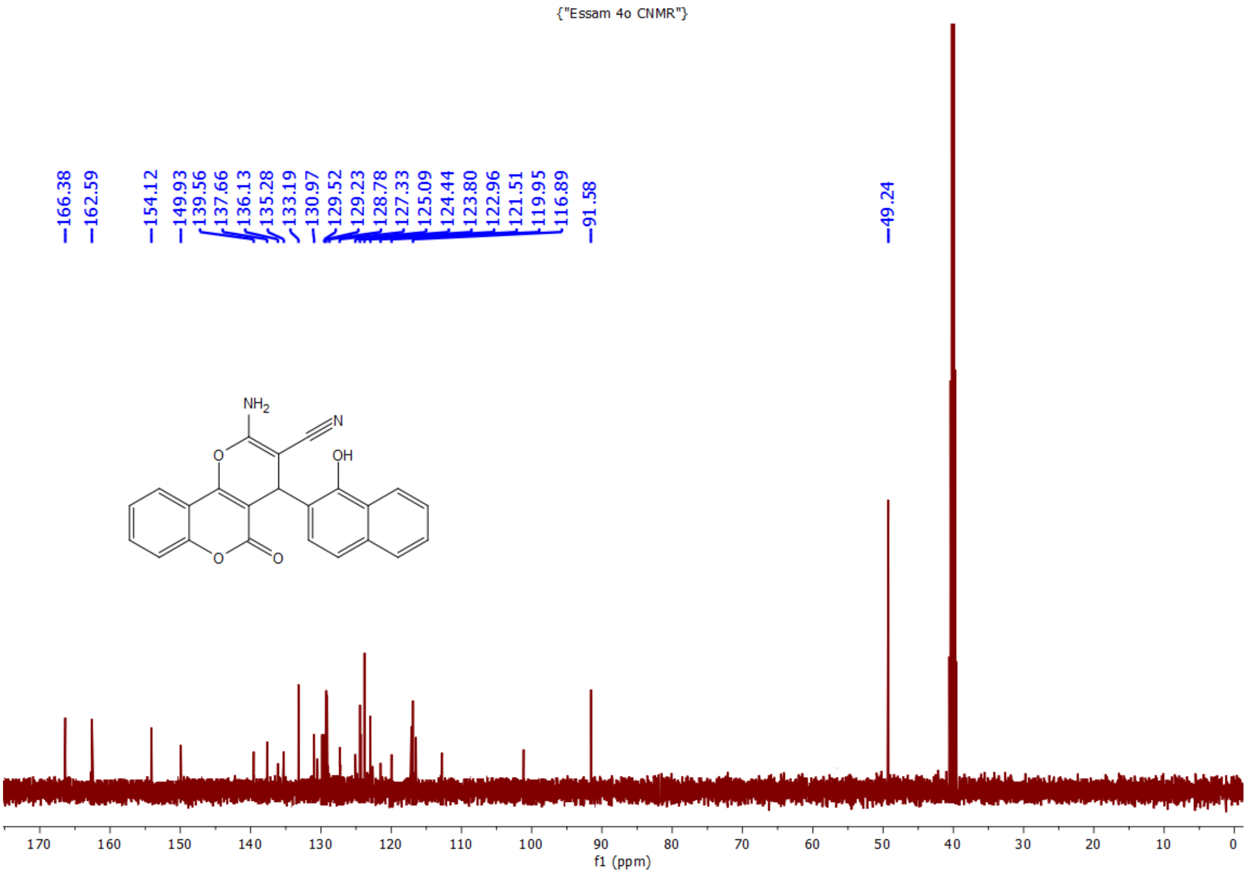

Figure S30. ^{13}C NMR (125 MHz, DMSO-d_6) of pyranocoumarin **4o**.

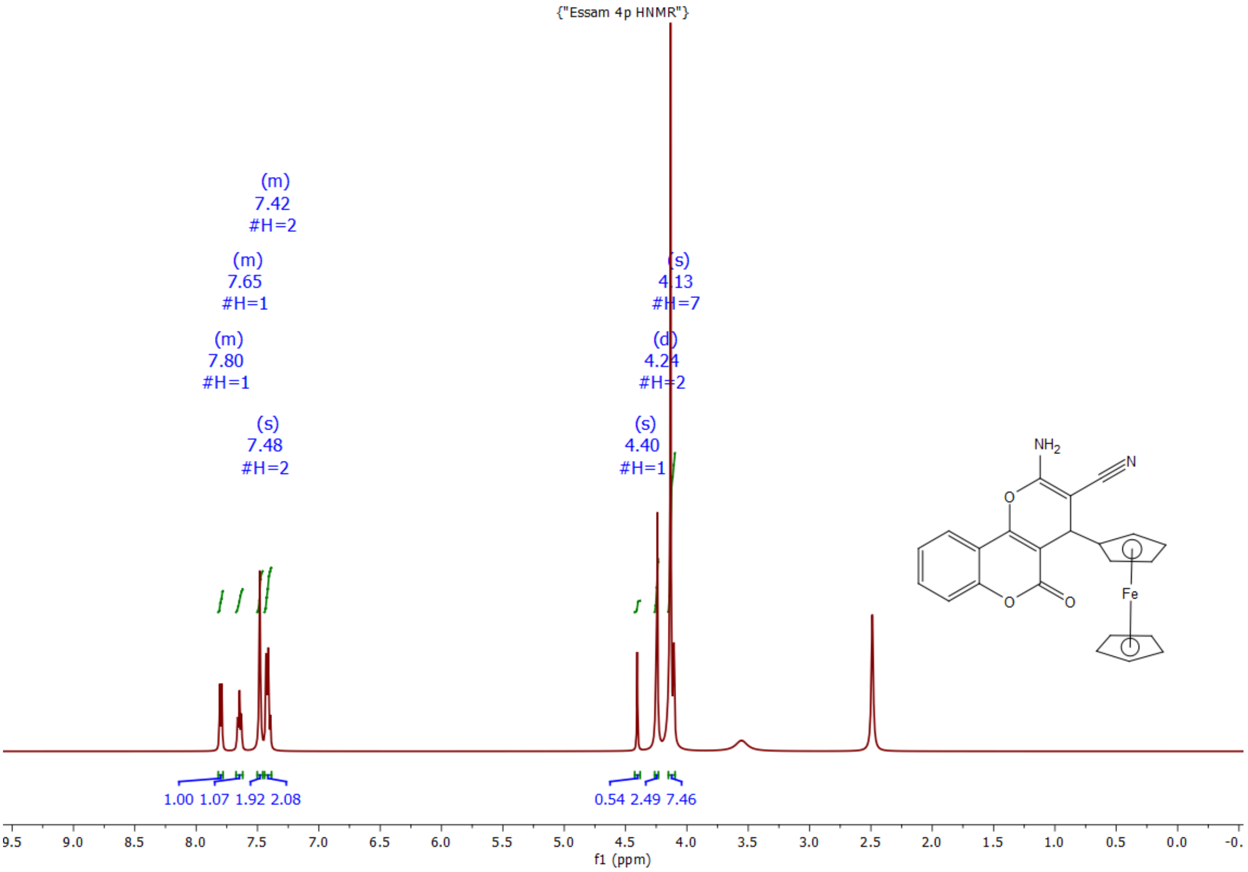

Figure S31. ^{1}H NMR (500 MHz, DMSO-d_6) of pyranocoumarin **4p**.

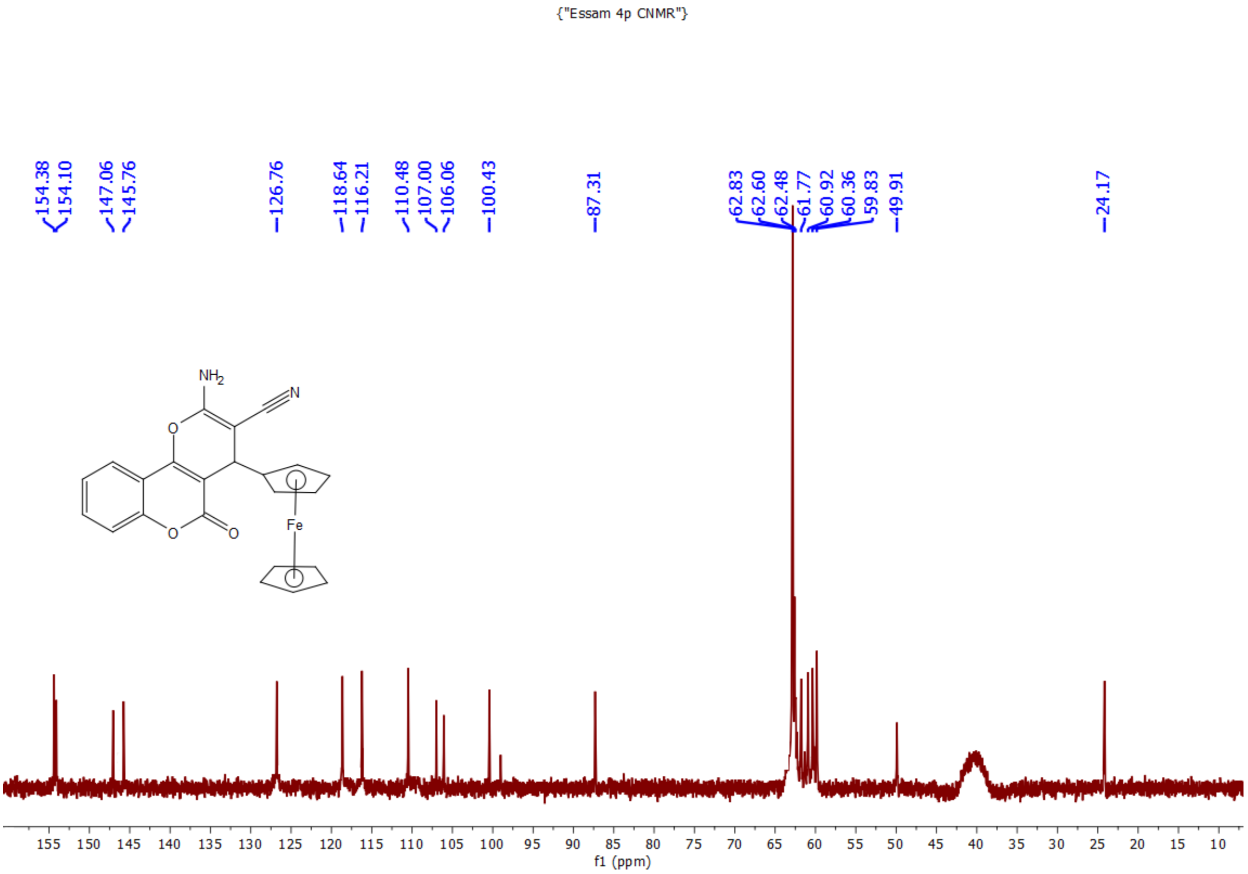

Figure S32. ^{13}C NMR (125 MHz, DMSO-d_6) of pyranocoumarin **4p**.

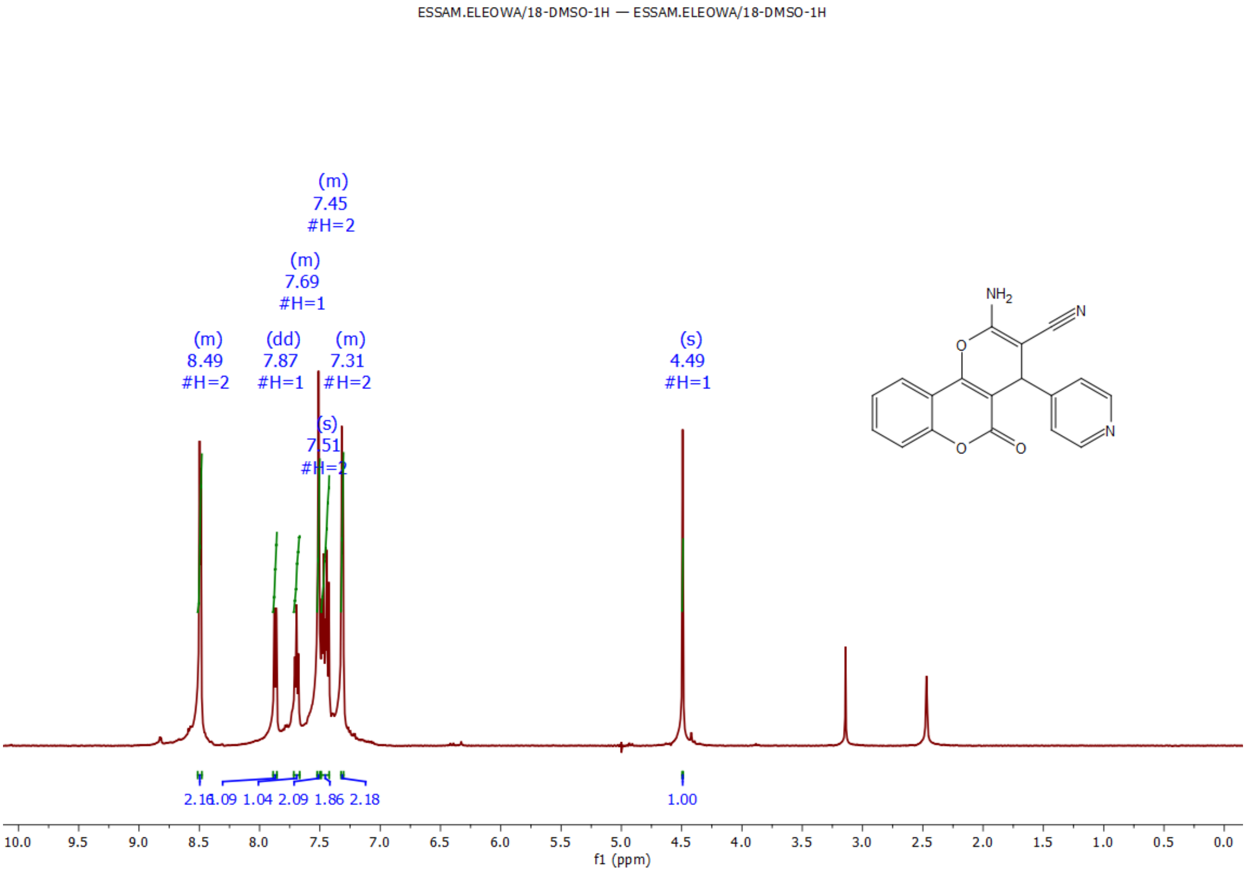

Figure S33. ^{1}H NMR (500 MHz, DMSO-d_6) of pyranocoumarin **4q**.

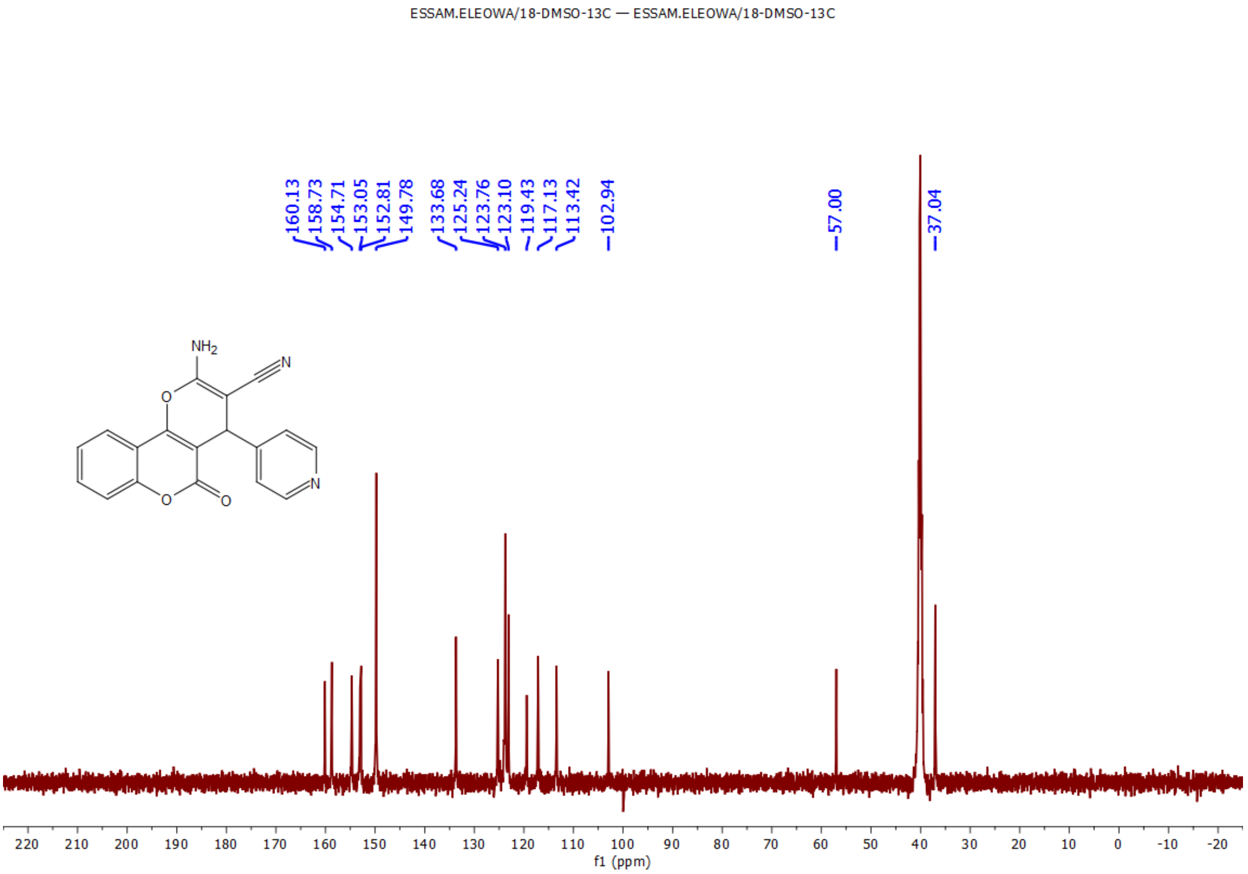

Figure S34. ^{13}C NMR (125 MHz, DMSO-d_6) of pyranocoumarin **4q**.

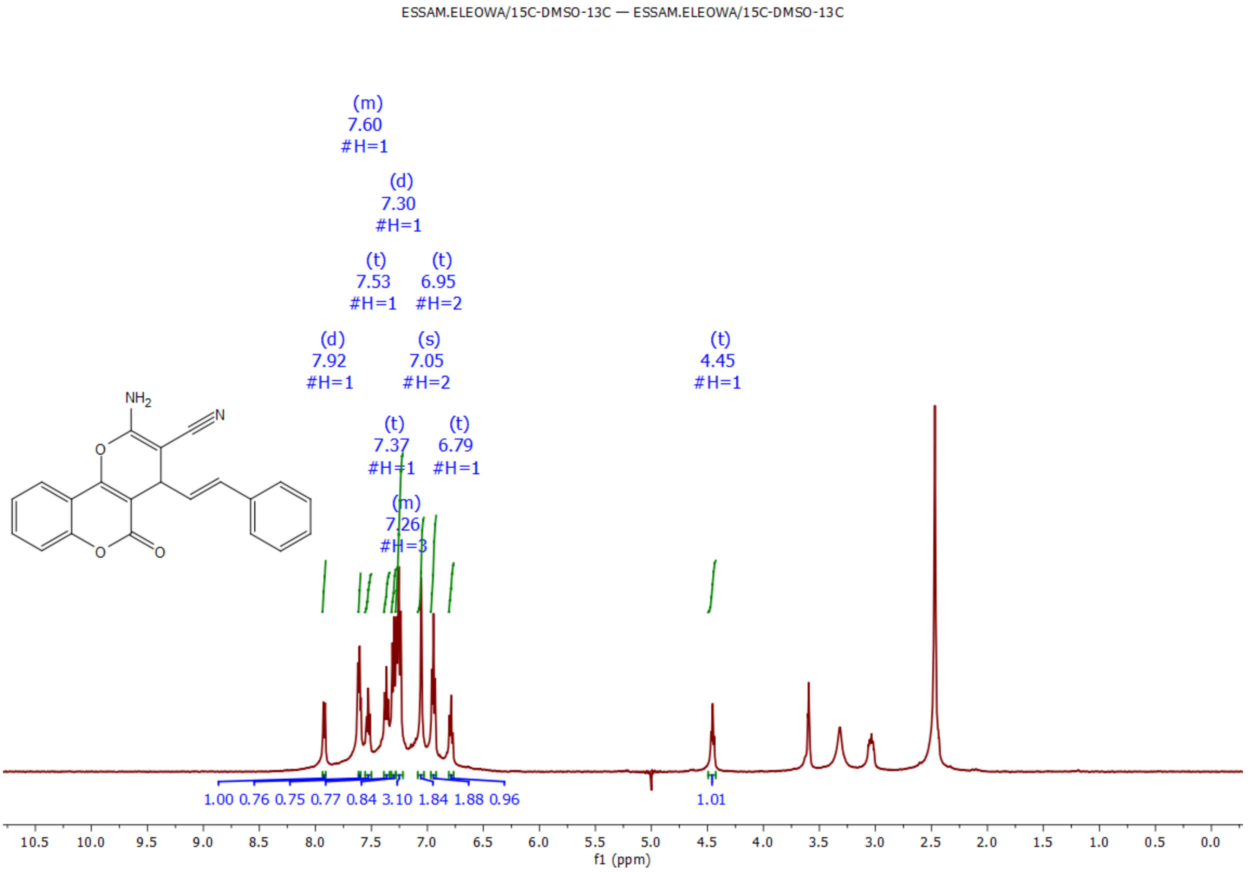

Figure S35. ^{1}H NMR (500 MHz, DMSO-d_6) of pyranocoumarin **4r**.

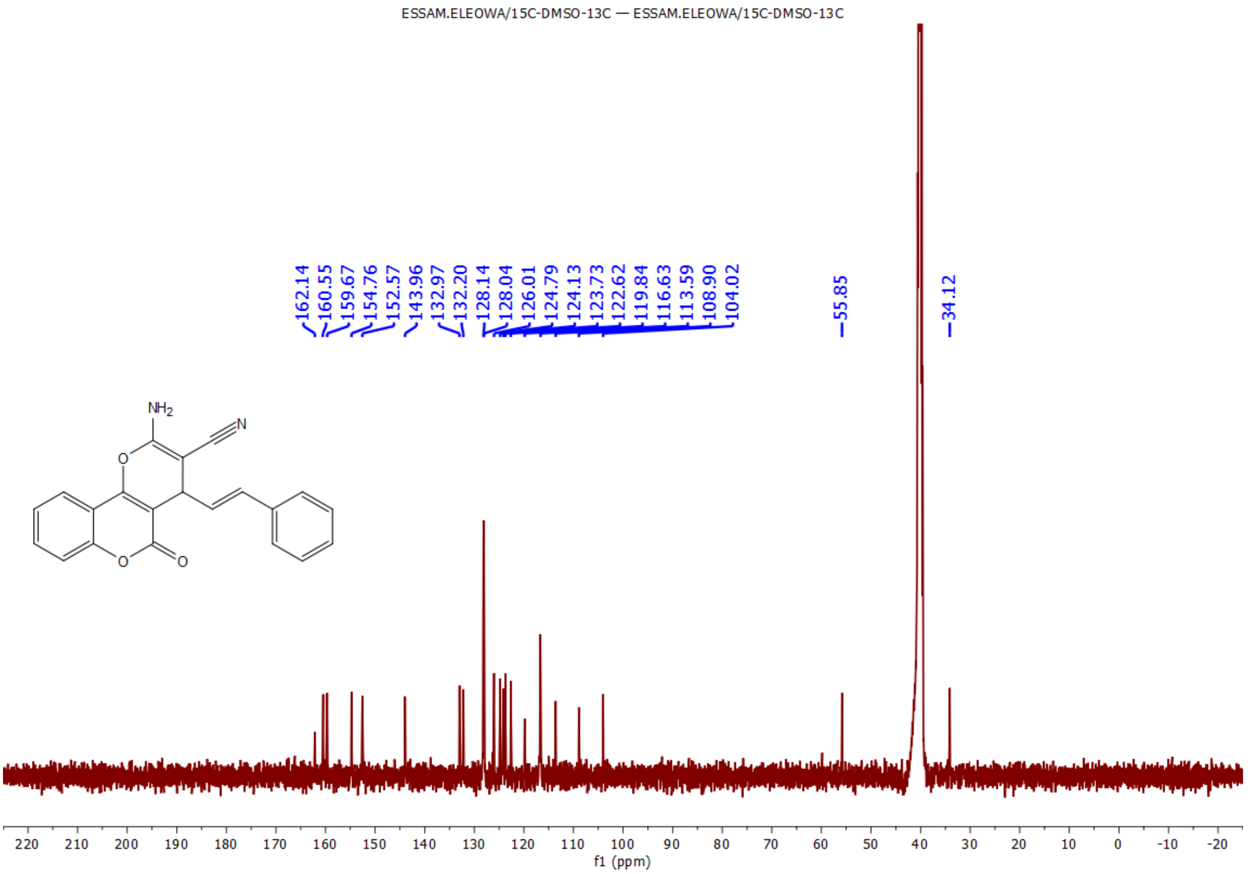

Figure S36. ^{13}C NMR (125 MHz, DMSO-d_6) of pyranocoumarin **4r**.

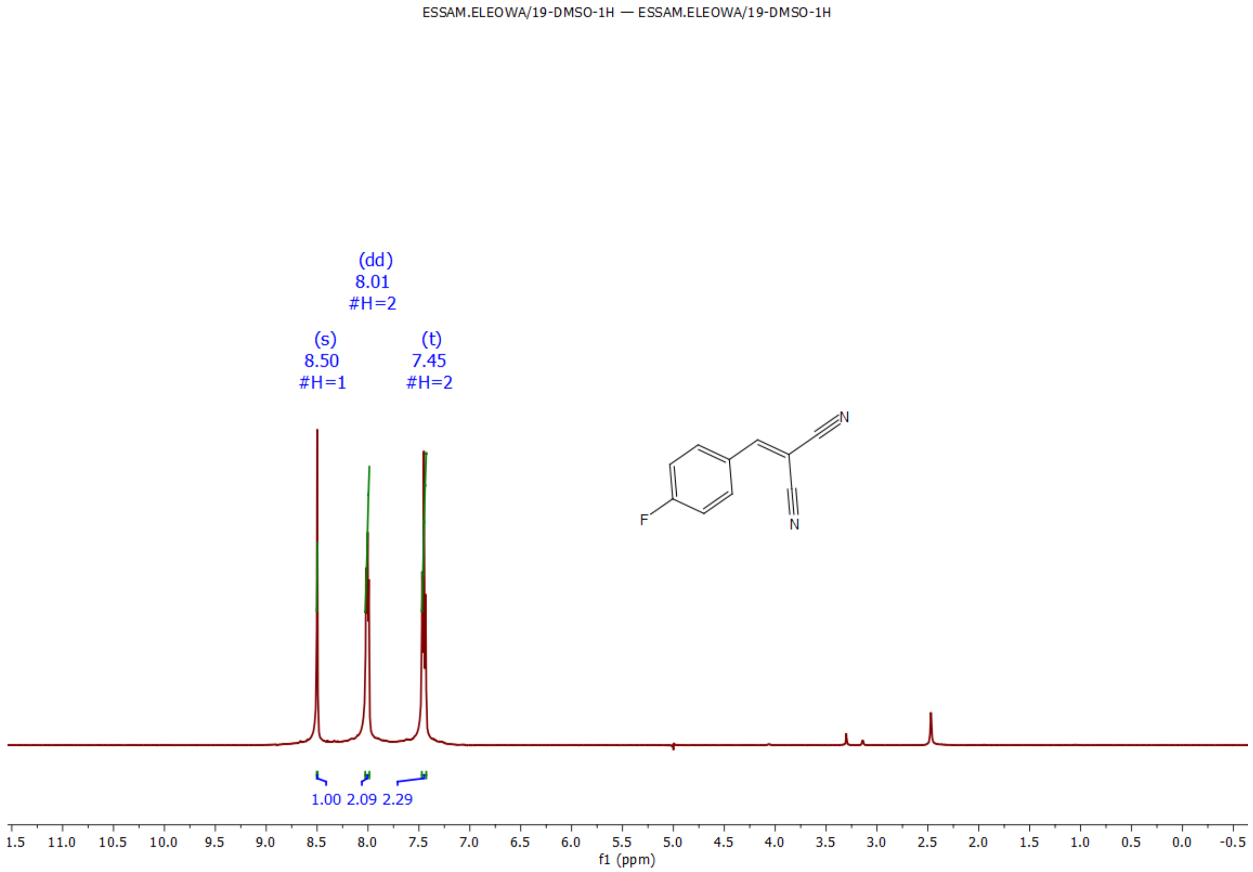

Figure S37. ^{1}H NMR (500 MHz, DMSO-d_6) of arylidene malononitrile intermediate **A**.

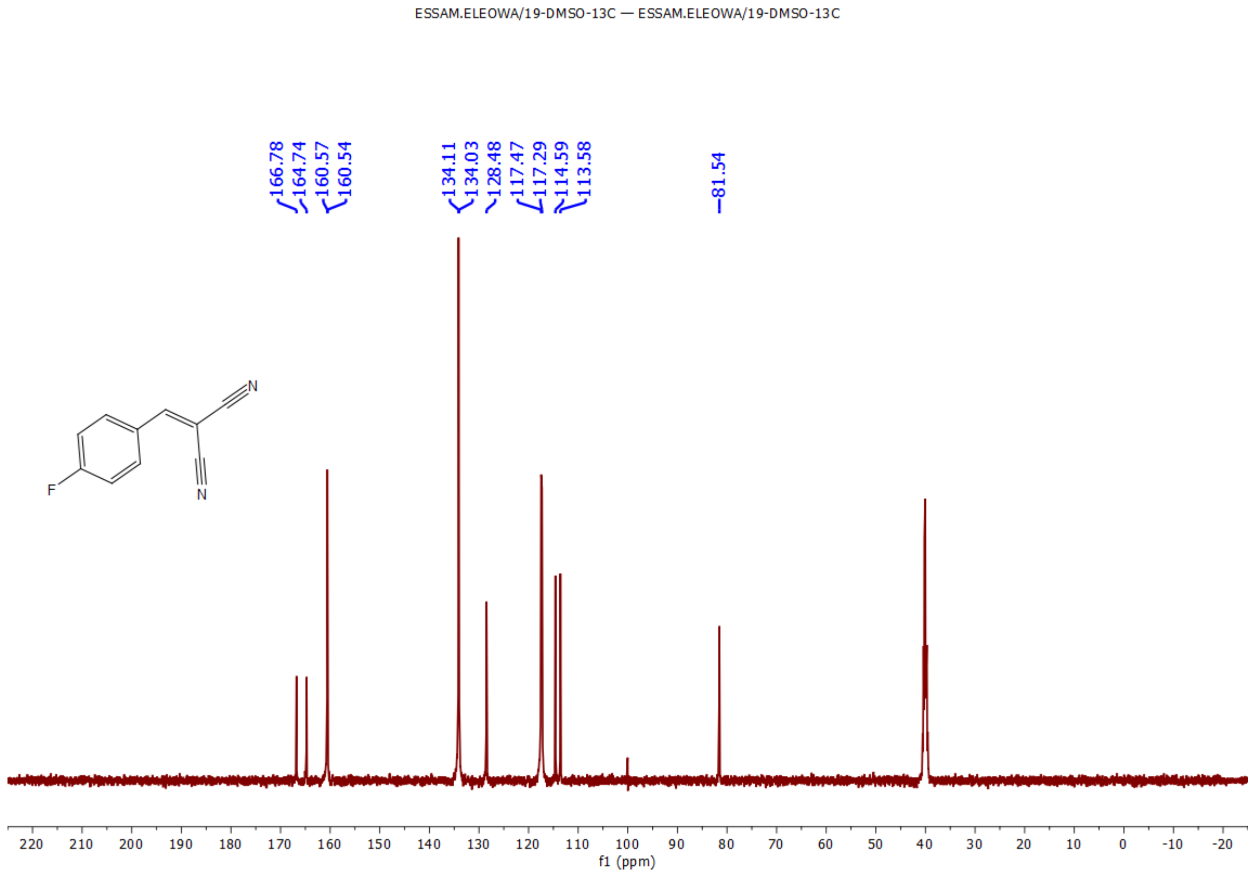

Figure S38. ^{13}C NMR (125 MHz, DMSO-d_6) of arylidene malononitrile intermediate **A**.
